# Nuclear Magnetic Resonance Chemical Shift as a Probe for Single‐Molecule Charge Transport

**DOI:** 10.1002/anie.202402413

**Published:** 2024-04-03

**Authors:** X. Qiao, A. Sil, S. Sangtarash, S. M. Smith, C. Wu, C. M. Robertson, R. J. Nichols, S. J. Higgins, H. Sadeghi, A. Vezzoli

**Affiliations:** ^1^ Department of Chemistry University of Liverpool Crown Street Liverpool L69 7ZD United Kingdom; ^2^ Device Modelling Group, School of Engineering University of Warwick Coventry CV4 7AL United Kingdom; ^3^ Institute of Optoelectronic Materials and Devices, Faculty of Materials Metallurgy and Chemistry Jiangxi University of Science and Technology Ganzhou 341000 China; ^4^ Stephenson Institute for Renewable Energy University of Liverpool Peach Street Liverpool L69 7ZF United Kingdom

**Keywords:** Molecular Devices, Molecular Electronics, NMR Spectroscopy, Quantum Chemistry

## Abstract

Existing modelling tools, developed to aid the design of efficient molecular wires and to better understand their charge‐transport behaviour and mechanism, have limitations in accuracy and computational cost. Further research is required to develop faster and more precise methods that can yield information on how charge transport properties are impacted by changes in the chemical structure of a molecular wire. In this study, we report a clear semilogarithmic correlation between charge transport efficiency and nuclear magnetic resonance chemical shifts in multiple series of molecular wires, also accounting for the presence of chemical substituents. The NMR data was used to inform a simple tight‐binding model that accurately captures the experimental single‐molecule conductance values, especially useful in this case as more sophisticated density functional theory calculations fail due to inherent limitations. Our study demonstrates the potential of NMR spectroscopy as a valuable tool for characterising, rationalising, and gaining additional insights on the charge transport properties of single‐molecule junctions.

## Graphical abstract

The chemical shift observed in NMR spectroscopy has been recently found to be a descriptor for the frontier molecular orbitals of organic/organometallic compounds. In this manuscript, we demonstrate that it can be applied to rationalise charge transport through such compounds, equipping the molecular electronics toolbox with another useful technique.

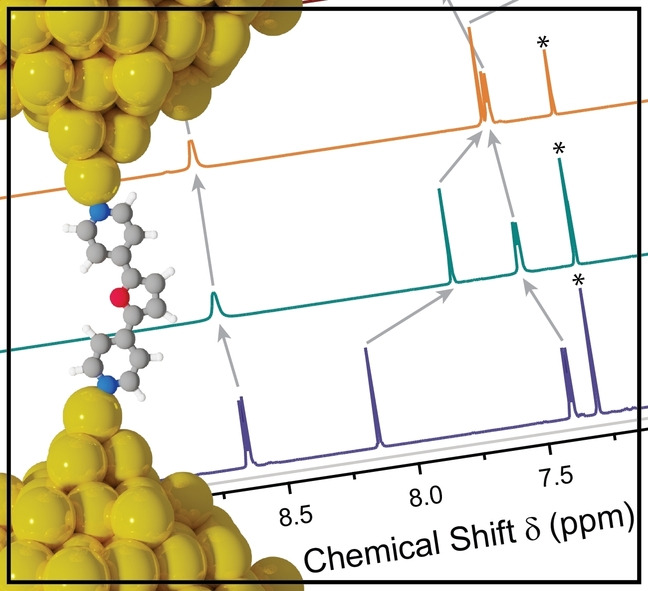

## Introduction

Single‐molecule electronics—the use of single molecules as electronic components—has evolved rapidly from a scientific curiosity into a useful tool to characterise interfacial effects,[^1^, ^2^, ^3^, ^4^] chemical reactivity at the single‐entity scale,[^5^, ^6^, ^7^] light‐matter interactions,[^8^, ^9^, ^10^] and mechanochemical phenomena.[^11^, ^12^, ^13^] Underpinning all these applications is the ability of a single molecule to allow transport of current as quantified by its single‐molecule conductance. Over the years, a wide range of models have been developed to aid the design of efficient molecular wires and to understand the physico‐chemical relationships determining the final charge transport properties. These models range from simple and intuitive translation of chemical rules (e.g. Hammett parameters,^[14]^ curly arrows,^[15]^ aromaticity[^16^, ^17^]), to qualitative theoretical models[^18^, ^19^] and quantitative density‐functional theory (DFT) computations.[^20^, ^21^, ^22^, ^23^] All these methods, however, present some limiting drawbacks. Simple chemical rules do not always translate directly to the single‐entity scale^[24]^ and, as we recently reported, the occurrence of internal charge reorganisation requires a holistic assessment of the entire molecular wire rather than the properties of its individual components.^[4]^ Qualitative theoretical methods, while useful, are not accurate,^[25]^ while full DFT calculations offer increased accuracy at greatly inflated computational cost, complicated by inherent errors^[26]^ and limitations.^[27]^ Further research is clearly needed to refine the simple, intuitive concepts that drive the initial design of molecular wires, and to develop faster but accurate modelling methods that can give insights on the physicochemical phenomena underpinning charge transport efficiency, with the final aim of establishing a structure–property relationships framework.

**Figure 1:**
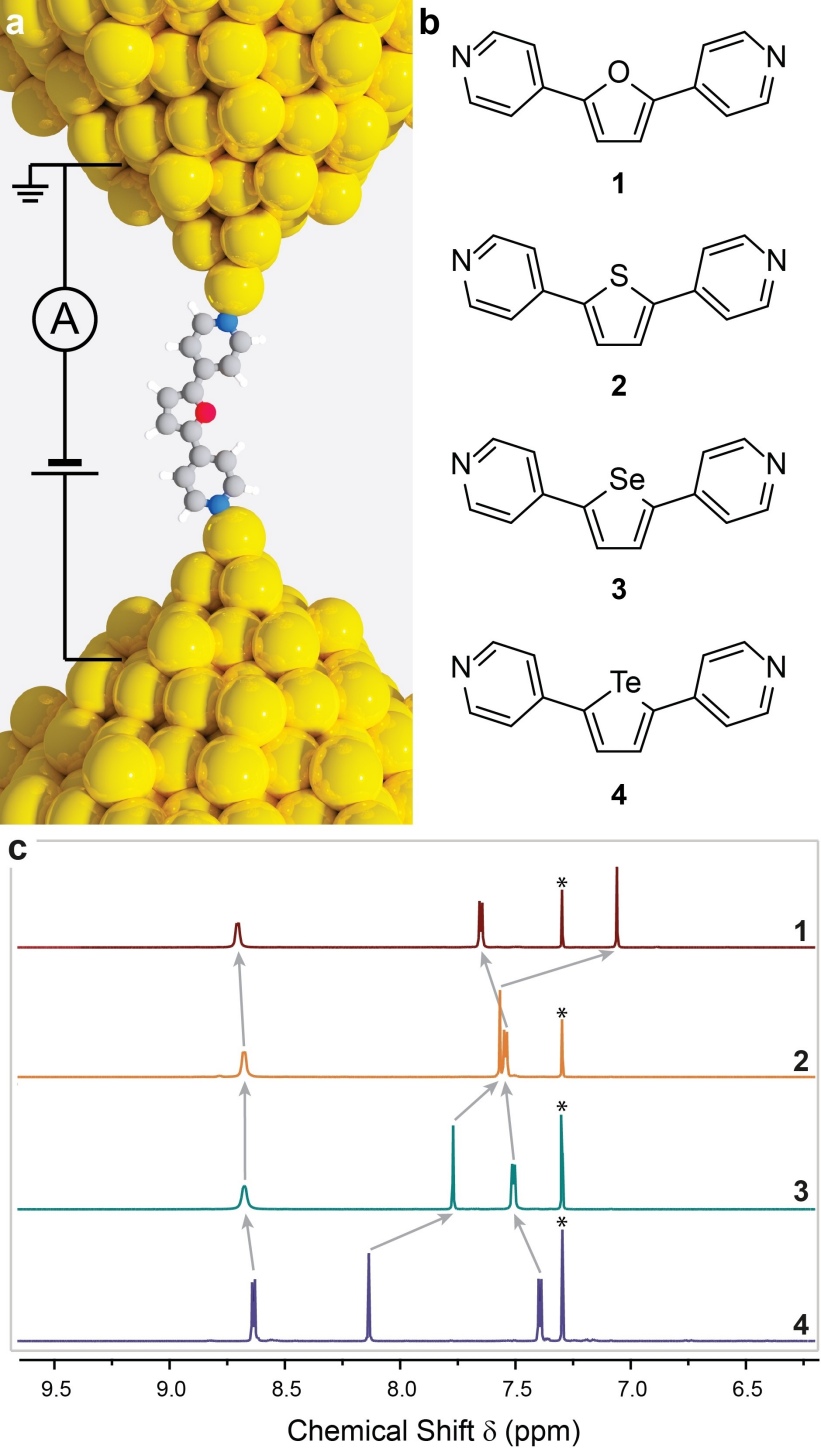
(a) Depiction of a single‐molecule junction, showing compound **1** assembled between two atomically sharp Au electrodes. (b) Structures of the molecular wires used in this study. (c) Expansion of the aromatic region of the ^1^H NMR spectra of **1**–**4**. Trends are highlighted with arrows in the spectra. The residual solvent resonance (CDCl_3_) is starred. Colour legend in (b): C=grey, N=blue, O=red, H=white, Au=yellow. NMR spectra in (c) obtained at 500 MHz in CDCl_3_.

Nuclear Magnetic Resonance (NMR) spectroscopy is a technique routinely used for the structural determination of organic and organometallic compounds. Non‐zero spin nuclei (e.g. ^1^H, ^13^C) are excited by radiofrequency radiation while subject to a strong magnetic field, and the displacement of the resonance frequency of nuclei is measured relative to that of a reference compound, as the chemical shift δ . Importantly, the chemical shift of a specific nucleus is indicative of its electronic environment, with both short‐ and long‐range^[28]^ effects contributing to its value. The chemical shift tensor has been demonstrated to be, for instance, a descriptor for the electronic structure of organic^[29]^ and organometallic^[30]^ compounds, providing information on the nature of their frontier molecular orbitals and possible catalytic reactivity.[^31^, ^32^] As such, we reasoned that NMR spectroscopy could be used as a probe for the electronic *transport* properties of single‐molecule junctions (Figure 1a), given that the electronic structure of the molecular wire, which determines the energy and character of the frontier orbitals, is indeed one the main factors affecting the transmission function (the probability of one electron to tunnel from the source to the drain electrode through the molecule).

In what follows, we demonstrate correlation between charge transport efficiency and chemical shift δ (^1^H/^13^C) in a series of molecular wires, along with additional data extracted from the literature that showcases the wide applicability of our method. Persistent convergence issues with DFT transport calculations of junctions incorporating heavy chalcogen atoms (e.g. Se, Te) prevented us from performing such calculations on the entire series of molecules presented here. We therefore used data extracted from the NMR spectra and additional magnetic calculations to inform and build a tight‐binding model which correctly interprets the data and highlights a previously unreported quantum interference phenomenon in heavy chalcogenophene‐containing molecular junctions.

## Results and Discussion

We focussed our initial efforts on synthesising the entire chalcogenic (group 16) metalloles, the chalcogenophenes, substituted in the 2,5‐positions by 4‐pyridyls as metallophilic electrode contacts (**1**–**4** in Figure 1b). These compounds are ideal for our study because they have clearly defined NMR signals, one of which (the singlet in Figure 1c, arising from the two equivalent protons on the chalcogenophene) has large changes in δ across the series.

We synthesised **1**–**4** (see the Supporting Information for methods, detailed procedures and characterisation) and used the *scanning tunnelling microscope—break junction* (*STMBJ*) technique^[33]^ to fabricate molecular junctions and measure their charge transport properties. In this technique, a Au STM tip is driven into a Au substrate under a DC bias (100mV in this study) to fabricate a microcontact of conductance $\gg \;G_{0}$ , where $G_{0}$ is the quantum of conductance $2e^{2}/h≅77.48\;\mu S$ . The tip is then withdrawn at constant speed ($12\;nm\;s^{-1}$ ) in a solution of the target molecule (1 mM in mesitylene:tetrahydrofuran 4 : 1). The electrical current is continuously monitored during the process, and the conductance is then calculated as G=I/V . As the tip is withdrawn, the microcontact is thinned to an atomic point contact, having $G=G_{0}$ , which is broken upon further withdrawal to yield two atomically sharp nanoelectrodes. Molecules present in solution or preadsorbed on the two electrodes^[34]^ can self‐assemble in the freshly prepared nanogap to fabricate a single‐molecule junction. The tip is further withdrawn to stretch the junction to its maximum length, until it is ruptured. The tip is then driven into the sample to fabricate a fresh metallic microcontact and the whole process is repeated thousands of times to collect a statistically robust dataset (>4000 individual junctions). All data obtained with this procedure are then compiled, with no further selection, into histograms and 2D maps that show the distribution of conductance values as a function of $G_{0}$ , available as internal calibration. A brief description of the materials and equipment used in this study is available in the methods section, and further details are available in our previous publications.[^35^, ^36^]

**Figure 2:**
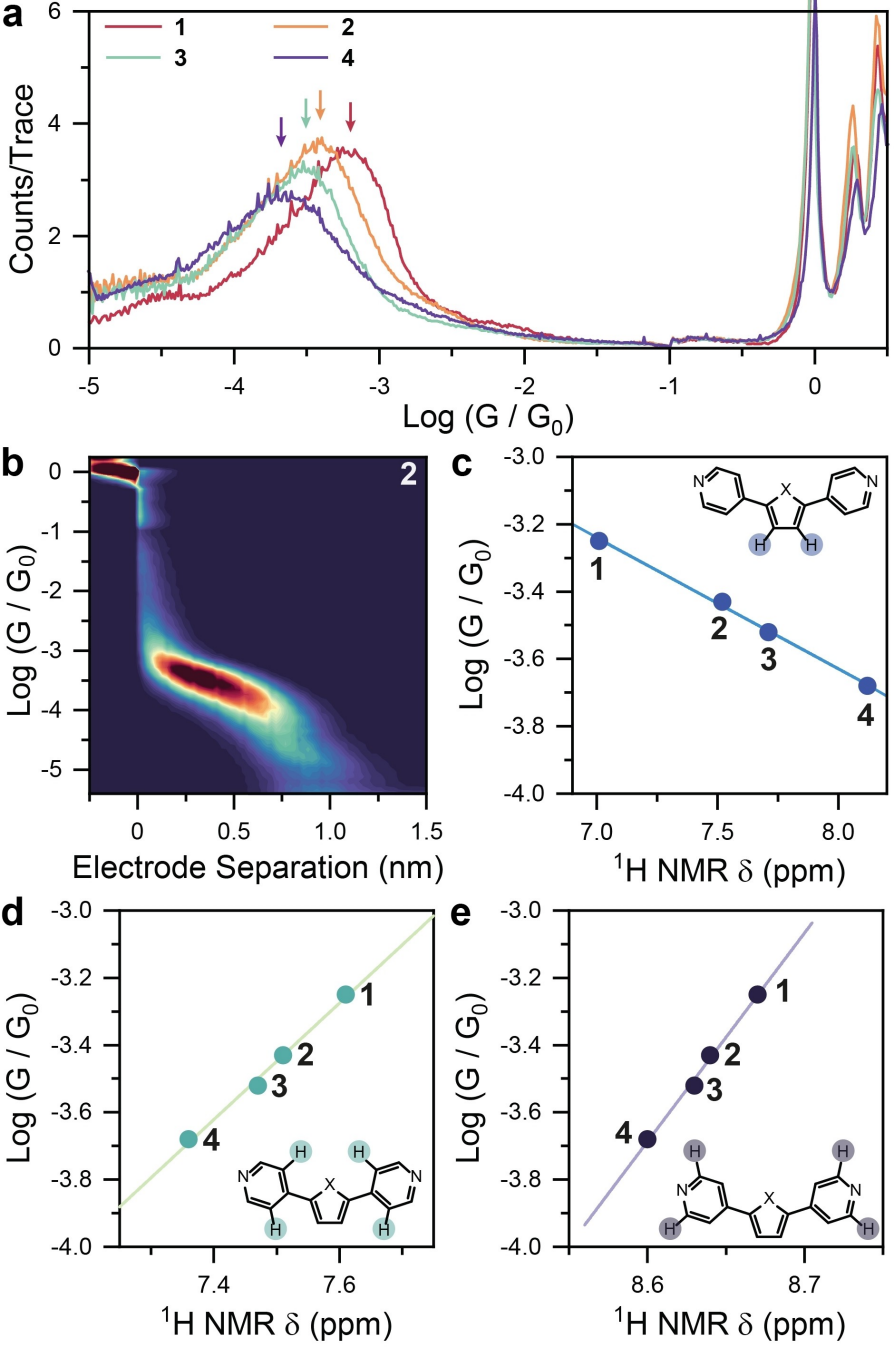
(a) *STMBJ* conductance histograms for compounds **1**–**4**. (b) 2D density map for **2**. Number of *STMBJ* traces used for the histograms in (a): **1**=4258; **2**=4784; **3**=5188; **4**=7803. (c–e) Conductance *vs* 1H NMR resonance position plots for **1**–**4**, with line fitting as guide to the eye. The proton responsible for the NMR resonance is explicitly shown and highlighted in the inset structure. (a) and (b) compiled with 100 bins / decade and 100 bins / nm. All data acquired at 100 mV bias. *STMBJ* experiments performed with the target compound present as 1 mM solution in mesitylene:tetrahydrofuran 4 : 1.

Our results show a clear trend, with conductance decreasing through the chalcogenophene series **1→4** (Figure 2a). Analysis of the two‐dimensional plots, where conductance is plotted against the electrode separation (an example for **2** is shown in Figure 2b, and those for **1**, **3**, and **4** can be found in the SI), along with further analysis of the conductance at the most statistically robust electrode separation, confirm that transport happens from one 4‐pyridyl termini to the other, with the potentially aurophilic atom of the chalcogenophene (e.g. S, Se, Te*)* not acting as ancillary/supporting contact^[37]^ to the electrodes. Quite strikingly, a semilogarithmic correlation of single‐molecule conductance with the ^1^H NMR chemical shift of the protons on the chalcogenophene (Figure 2c), and with the ^13^C{^1^H} NMR chemical shift of the carbon in the β ‐position with respect to the chalcogen (see Supporting Information for details). The chemical shifts δ of the pyridyl protons are also clearly correlated with conductance (Figure 2d–e), but the small magnitude of the change (cf. with spectra in Figure 1c) suggests only modest alterations in the electronic structure of the pyridyl ring across the **1**–**4** series. We therefore focussed our attention in the following experiments and data interpretation on the chalcogenophene β‐proton resonance, where the large variations in δ observed suggest significant changes in the electronic environment reflected in their charge‐transport properties.

We can discount an interpretation of our data based on pure aromaticity decreasing charge transport efficiency as found by Chen et al.^[16]^ Spectroscopic and reactivity studies have established the order of aromaticity as furan< tellurophene≪ selenophene≤ thiophene< benzene,^[38]^ with theoretical calculations also confirming selenophene and thiophene as having stronger aromatic stabilisation than tellurophene and furan.[^39^, ^40^] This order matches that of reactivity towards electrophilic substitution (e.g. formylation, acetylation), which is furan> tellurophene≫ selenophene> thiophene> benzene,^[41]^ or that of reactivity in Diels–Alder cycloaddition (which can be used as a proxy for the diene character of the chalcogenophene) that is furan≫ selenophene> thiophene,^[42]^ with tellurophene also displaying remarkable reactivity to Diels–Alder conditions.^[43]^ Therefore, the trend of single‐molecule conductance of **1**–**4** ($G_{tellurophene}<G_{selenophene}<G_{thiophene}<G_{furan}$ ) does not follow the aromaticity order. Further discussion of the aromaticity of **1**–**4** based on their structural parameters can be found in the SI.

Similar arguments can be applied to the analysis of the NMR spectra to understand the origin of the observed significant deshielding of the chalcogenophene protons (singlet in Figure 1c). While it is tempting to attribute it to aromatic ring currents, a naïve interpretation that led the authors of the first synthesis of tellurophene^[44]^ to propose a marked aromatic character, recent calculations of nucleus‐independent chemical shifts,^[40]^ show the opposite is true: ring currents in tellurophene (and furan) are significantly weaker than in selenophene and thiophene.^[45]^ We can also discount the electronegativity of the heteroatom being responsible for the change in δ , as the most electronegative element (O) should contribute the strongest deshielding (shift of δ to more downfield values), the opposite of the observed trend. We therefore carried out DFT calculations to estimate the Chemical Shift Tensor (CST) components on the protons of the chalcogenophene and the overall degree of anisotropy (e.g. the deviation of the three component of the tensor from their average, isotropic value). It has been demonstrated that the CST components are related to the symmetry and energy of the frontier orbitals, and they are strongly influenced by the electronic structure of the molecule of interest.^[29]^ DFT simulations were able to reproduce the experimental trend, and numerical results of our theoretical investigation are reported in the SI. The degree of anisotropy of the CST increases across the series, suggesting that the chalcogen has a strong influence on the electronic structure of the π‐system of the molecular wire. This hypothesis is strengthened by the analysis of the principal components of the tensor (see Supporting Information for details) showing that, while $\sigma_{33}$ is consistently aligned out‐of‐plane, $\sigma_{22}$ increases its orientation towards the chalcogen as we go across the **1**–**3** series, with good correlation with single‐molecule conductance. The magnitude of $\sigma_{11}$ , on the other hand, does not follow a clear trend in **1**–**3**, and its varying orientation, roughly aligned with the pyridyl‐pyridyl axis, suggests that the geometric distortions induced by the increasing size of the chalcogen do not have a significant effect on the electronic structure of the molecular wire.

As first attempt at modelling the transport phenomena, we performed transport calculations using the GOLLUM transport code,[^21^, ^46^] combined with the mean‐field Hamiltonian calculated using the SIESTA^[20]^ implementation of DFT. Due to the computational limitations of DFT, and in particular, persistent convergence issues when dealing with junctions containing heavy atoms (**3** and **4**), the transmission coefficient could only be calculated for compounds **1** and **2**. The results are presented in Figure S28 of the SI, which are in agreement with the experimental trend (${\;G}_{thiophene}<G_{furan}$ ).

**Figure 3:**
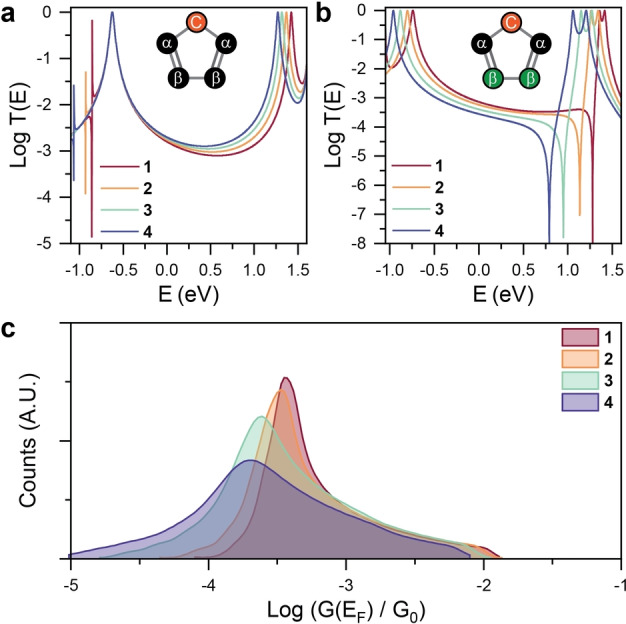
Theoretical modelling. (a) Tight‐binding transmission curves for 5‐membered ring system when only on‐site energy of site C $\epsilon_{C}$ changes to −0.6, −0.8, −1, and −1.2 for O, S, Se, and Te, respectively. (b) Transmission curves for the structures when $\epsilon_{C}$ changes from −0.6, −0.8, −1, and −1.2, and $\epsilon_{\beta }$ is −0.48, −0.64, −0.83 and −1, for O, S, Se, and Te, respectively. (c) Calculated conductance histograms for the TB model in (b) obtained by randomising the on‐site energies in the range ±0.2eV .

In order to overcome the limitations imposed by DFT, we constructed a tight‐binding (TB) model in which a single *p*‐orbital per atom only interacts with its nearest neighbor orbitals, with coupling integrals γ=-1eV . We started by setting the on‐site energies for all carbon atoms ($\epsilon_{\alpha }$ and $\epsilon_{\beta }$ ) to zero in the TB Hamiltonian, and we then calculated the transmission function *T(E)* between α sites (see the structure shown in the inset of Figure 3a). By doing this, we only consider the effect of the chalcogen (orange TB site C in Figure 3) on its neighbour sites α , and the overall trend we found is opposite to the experimental results: the conductance increases as the on‐site energy $\epsilon_{C}$ is changed from −0.6 for O (**1**) to −1.2 for Te (**4**, Figure 3a). However, our NMR experimental results indicate large changes to the electronic environment of the β atoms (green sites in Figure 3b) in the **1→4** series, suggesting that the potential profile of these sites is heavily affected by the increasing ability of the chalcogen to perturb the π ‐system. To simulate this effect, we applied non‐zero values also to the on‐site energies of the β atoms ($\epsilon_{\beta }$ ) of our model. Importantly, the values of $\epsilon_{\beta }$ were chosen using the CST calculations as guideline for their relative magnitude, to ensure consistency between the two methods. Using these changes to the TB Hamiltonian, we then re‐calculated the transmission coefficient (Figure 3b), obtaining now a trend in good agreement with the experiments, with the transmission amplitude decreasing as the $\epsilon_{\beta }$ values are changed. Furthermore, we see changes in the quantum interference pattern from constructive interference (Figure 3a) to destructive interference (Figure 3b), which are the hallmark of a chalcogen‐induced disturbance to the conjugated π ‐system. We then randomized the potentials of the on‐site energies $\epsilon_{C}$ and $\epsilon_{\beta }$ in the ±0.2eV range, and we calculated the transmission coefficient for each of the resulting Hamiltonians (see Supporting Information for further details). The process is intended to verify the robustness of our model against the small changes to the environment that molecular junctions experience during a measurement (e.g. nearby molecules, solvents, thermal vibrations). Using the obtained transmission coefficients, we calculated the room‐temperature electrical conductance using the Landauer formula and compiled the resulting values in conductance histograms using a method we previously established.[^47^, ^48^] The theoretical histograms in Figure 3c are in a good agreement with the experimental ones shown in Figure 2a, both in terms of conductance and in overall shape of the histogram (e.g. **4** displaying a broader conductance distribution than **1**). These results show the resilience of the transmission trend to the small variations in the Hamiltonian and the significant role of $\epsilon_{\beta }$ , and hence the perturbation of the π‐system induced by heavy chalcogens, in determining the charge transport properties of the system.

**Figure 4:**
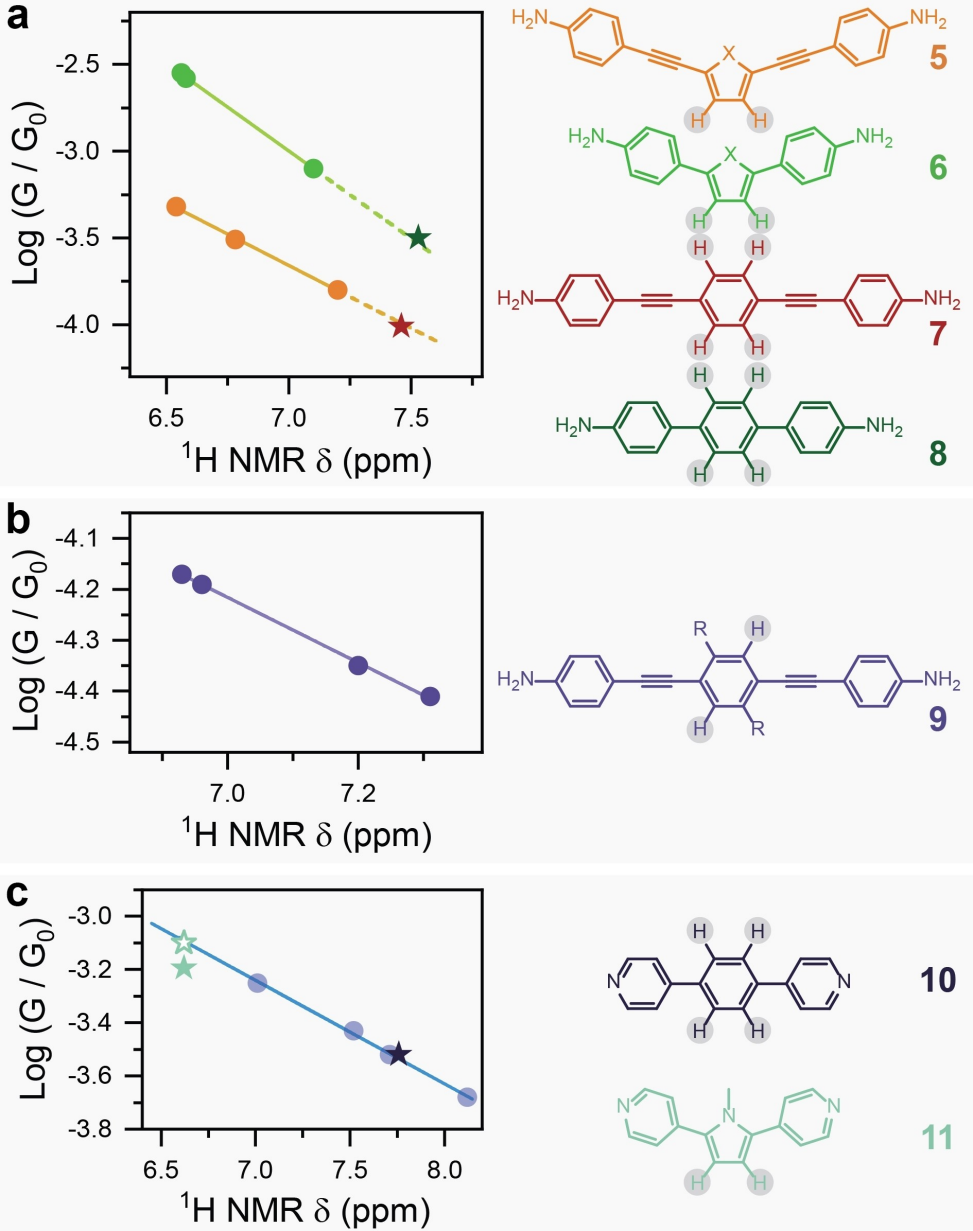
Correlation of single‐molecule conductance and ^1^H NMR δ . (a) Examples taken from Chen et al.^[16]^ (**5** and **6**, circles, X=S, O, CMe_2_), and phenyl derivatives (**7** ^[49]^ and **8**,^[50]^ stars). ^1^H NMR data obtained in DMSO‐d6, *STMBJ* measurements performed in 1,2,4‐trichlorobenzene. (b) Examples taken from González et al.^[51]^ demonstrating sensitivity to molecular wire substituents R=H, *n‐*octyl, OCH_3_, and OC_6_H_13_) on the OPE core **9**. ^1^H NMR data obtained in CDCl_3_, *STMBJ* measurements performed in 1,2,4‐trichlorobenzene. (c) Extension of the **1**–**4** series presented in this manuscript (circles) to include phenyl‐ and pyrrole‐containing molecular wires (**10** ^[50]^ and **11**, stars). 1H NMR data obtained in CDCl_3_, *STMBJ* measurements performed in mesitylene:tetrahydrofuran 1 : 4 v : v. Proton environments chosen to showcase the correlation explicitly shown in the structure of the molecular wires.

Having established the physical phenomenon underpinning the agreement between NMR chemical shifts and charge transport efficiency, we turned our attention to verify the robustness of the correlation between NMR chemical shift values and single‐molecule charge‐transport efficiency. To do this, we searched the published literature for examples of molecular wires that could show clear peaks with straightforward assignment (e.g. not in need of 2D NMR techniques), and with accompanying data from both techniques available for digitisation and plotting. While, ideally, *STMBJ* experiments and NMR spectra should be acquired in the same medium, we found that consistent experimental conditions (e.g. NMR spectra acquired in the same solvent, *STMBJ* performed in the same environment, etc.) were still able to return excellent correlation. Results of our survey are shown in Figure 4.

Data acquired on aniline‐terminated molecular wires (hence *p*‐type, as opposed to the *n*‐type 4‐pyridyl termini^[52]^ we synthesised) also showed excellent correlation between chemical shift δ and single‐molecule conductance. The five‐membered ring systems **5**–**6** ^[16]^ provided a stable benchmark line that could be extrapolated to the phenyl‐containing oligophenyleneethynylene (OPE) derivative **7** ^[49]^ and the terphenyl compound **8** ^[50]^ (Figure 4a). Excellent correlation was also found when chemical substitution is employed to modulate conductance, as demonstrated in Figure 4b for the OPE derivatives **9**.^[51]^ It is important to stress here that Figure 4a and Figure 4b cannot be directly compared as the NMR measurements were performed in different environments (DMSO‐d6 and CDCl_3_, respectively). Excellent agreement was also found between the trend line extrapolated from the pyridyl‐terminated system we investigated (**1**–**4**) and 1,4‐di(pyridin‐4‐yl)benzene **10** ^[50]^ (dark star in Figure 4c), thus further confirming the validity of the correlation even beyond 5‐membered ring systems.

Finally, we tested the limits of the pyridyl‐terminated system by synthesizing the pyrrole derivative **11** and measuring its single‐molecule conductance. Analysis of the NMR spectra show the singlet arising from the β protons of **11** resonating at 6.56 ppm, which according to the model discussed earlier and shown in Figure 2c would deliver a single‐molecule conductance value of $10^{-3.11}\;G_{0}$ . Measurements performed with the *STMBJ* technique returned a value of $10^{-3.2}{\;G}_{0}$ (See Supporting Information for synthesis, characterisation and *STMBJ* experiments). The small discrepancy (see Figure 4c, experimental value in filled teal star and predicted value in empty teal star) can be attributed to (i) the switch from a chalcogen to a pnictogen and the presence of a substituent on the pyrrolic N, which introduces small geometric deformations unaccounted for by NMR spectroscopy but reflected in the observed charge‐transport efficiency, (ii) the presence of a methyl substituent on the pyrrolic N, which disrupts conjugation by twisting the pyrrole ring out‐of‐plane with the 4‐pyridyl termini, or (iii) the approach of the system to its conductance upper ceiling, where the electronic transparency of the pyridyl molecule/electrode interface dominates over transport through the backbone. The latter interpretation is supported by the fact that 4,4′‐dipyridyl (i.e. **11** without the 5‐membered ring) has conductance of ($10^{-3.15}\;G_{0}$ ),^[11]^ and we were unfortunately unable to synthesise the analogue of **11** without the methyl substituent. Nevertheless, **11** has higher conductance than **1**, and the trend of conductance agrees with the NMR δ , thereby confirming the β‐protons acting as a local probe for charge transport efficiency.

## Conclusion

In brief, we found that single‐molecule conductance and nuclear magnetic resonance chemical shifts are intrinsically correlated in a semilogarithmic relationship, as expected from a quantum tunnelling transport mechanism through frontier orbitals of the molecular wire. The NMR data and theoretical modelling have been used to gain further insights on the charge transport profile, demonstrating that in the cases analysed here, a decrease in conductance and a shift of the NMR proton resonance peaks of the molecular wire are due to the same phenomenon: a disturbance to the electronic structure of the conductive π ‐system by a heavy chalcogen which introduces quantum interference features in the transmission profile. We further validated our method through several datasets obtained from the literature, demonstrating the robustness of the correlation. More work is indeed needed to gain a deeper understanding of the physical phenomena linking NMR spectroscopy and single‐molecule conductance. Studies of molecular wires lacking the desirable properties of symmetry and simple NMR spectra that made the correlation evident in this study would especially benefit from a framework of ground rules to determine the appropriate NMR resonances to use as probe. Furthermore, to have a real measure of the orbital asymmetry, it would be highly beneficial to compare single‐molecule conductance data with solid‐state NMR measurements. Nevertheless, our work provides a simple frame of reference and a straightforward way to acquire more information on the electronic structure of molecular wires, that can then be used to better understand their charge transport behaviour.

## Conflict of interests

The authors declare no conflict of interest.

1

## Supporting information

As a service to our authors and readers, this journal provides supporting information supplied by the authors. Such materials are peer reviewed and may be re‐organized for online delivery, but are not copy‐edited or typeset. Technical support issues arising from supporting information (other than missing files) should be addressed to the authors.

Supporting Information

## Data Availability

The crystal structure of **4** has been deposited on the Cambridge Crystallographic Data Centre (CCDC), deposition #2219092. Data acquired in Liverpool for this study (NMR and *STMBJ*) are available on the University of Liverpool Data Catalogue at DOI: https://doi.org/10.17638/datacat.liverpool.ac.uk/2141.
