# Supplementary material for: Nuclear Magnetic Resonance Chemical Shift as a Probe for Single‐Molecule Charge Transport
Source: Angew Chem Int Ed Engl. 2024 Apr 3;63(19):e202402413. doi: 10.1002/anie.202402413 (PMC11497234; doi:10.1002/anie.202402413)
Supplement: Supplementary file 1 — Supporting Information [file ANIE-63-e202402413-s001.pdf]

Angewandte Chemie
Eine Zeitschrift der Gesellschaft Deutscher Chemiker

Supporting Information

Nuclear Magnetic Resonance Chemical Shift as a Probe for Single-Molecule Charge Transport

X. Qiao, A. Sil, S. Sangtarash, S. M. Smith, C. Wu, C. M. Robertson, R. J. Nichols, S. J. Higgins, H. Sadeghi, A. Vezzoli**

Nuclear Magnetic Resonance Chemical Shift as a Probe for Single-Molecule Charge Transport

X. Qiao,[a,#] A. Sil,[a,#] S. Sangtarash,[b,#] S. M. Smith,[a] C. Wu,[a,c] C. M. Robertson,[a] R. J. Nichols,[a] S. J. Higgins,[a] H. Sadeghi[b] and A. Vezzoli[a,d]

a) Department of Chemistry, University of Liverpool, Crown Street, Liverpool L69 7ZD, United Kingdom

b) Device Modelling Group, School of Engineering, University of Warwick, Coventry CV4 7AL, United Kingdom

c) Institute of Optoelectronic Materials and Devices, Faculty of Materials Metallurgy and Chemistry, Jiangxi University of Science and Technology, Ganzhou 341000, China.

d) Stephenson Institute for Renewable Energy, University of Liverpool, Peach Street, Liverpool L69 7ZF, United Kingdom

#: these authors contributed equally to this work

Contents

1. Synthetic Procedures 3

1.1 Synthesis of 2,5-di(pyridin-4-yl)furan (1) 3

1.2 Synthesis of 2,5-di(pyridin-4-yl)thiophene (2) 4

1.3 Synthesis of 2,5-di(pyridin-4-yl)selenophene (3) 4

1.4 Synthesis of 2,5-di(pyridin-4-yl)tellurophene (4) 5

1.5 Synthesis of 2,5-dibromo-1-methylpyrrole 5

1.6 Synthesis of 1-Methyl-2,5-bis(4-pyridyl)pyrrole (11) 6

1.7 NMR Spectra 6

1.8 Crystallographic Characterisation of 4 12

2. STMBJ Methods and Additional Data 15

2.1 Histogram and Density Plot for compound 1 15

2.2 Histogram and Density Plot for compound 2 16

2.3 Histogram and Density Plot for compound 3 16

2.4 Histogram and Density Plot for compound 4 17

2.5 Histogram and Density Plot for compound 11 17

3. NMR – Conductance Correlation 18

3.1 Further Details on 1-4 18

3.2 NMR DFT calculations 18

3.6 Principal Components 19

3.7 Plateau Length Analysis and Correlation at the Most Common Junction Extension 22

4. Additional Considerations on the Aromaticity of 1-4 25

5. Theoretical Methods 26

5.1 Tight-Binding Calculations 26

5.2 DFT Calculations 26

References 28

1. Synthetic Procedures

All reactions were performed under inert atmosphere (Ar or N_2), in oven-dried glassware. Reagents were purchased from commercial vendors (Sigma-Aldrich, TCI UK or Fluorochem), and all solvents were purchased from ThermoFisher Scientific. All chemicals were used with no further purification except $Pd(PPh_3)_4$ which was washed with cold, dry methanol and dried *in vacuo* before use. Thin layer chromatography was performed on Merck Silica Gel 60 F-254 plates. Flash column chromatography was performed manually using technical grade Sigma-Aldrich silica (230-400mesh, 60 Å pore size) or with a Teledyne ISCO CombiFlash NextGen 300, using RediSep disposable columns (230-400mesh, 60 Å pore size). ^{1}H and $^{13}C\{^{1}H\}$ NMR were recorded on a Bruker Avance III 500 MHz spectrometer and referenced to internal TMS and residual solvent peak. Mass spectra were recorded with an Agilent Q-TOF 7200 spectrometer. **1, 2, 3,** and **11** were synthesised by Suzuki coupling of the relevant 2,5-dibromo substituted chalcogenophene with 4-pyridinylboronic acid. 2,5-dibromo-1-methylpyrrole was synthesise following the procedure originally developed by Faigl *et al.*[1] Due to the absence of 2,5-dibromotellurophene as starting material, **4** was instead prepared by treating 1,4-di(pyridine-4-yl)buta-1,3-diyne[2] with sodium telluride, obtained by *in situ* reduction of tellurium metal with $NaBH_4$.[3] All compounds were purified by chromatography followed by recrystallisation (reprecipitation in the case of **1**, which we were unable to obtain in crystalline form) to produce analytically pure samples.

No attempts were made at optimising yield.

1.1 Synthesis of 2,5-di(pyridin-4-yl)furan (1)

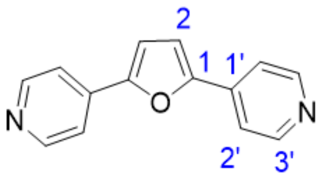

2,5-Dibromofuran (1.00 g, 4.43 mmol), 4-pyridylboronic acid (2.72 g, 22.15 mmol), potassium carbonate (6.12 g, 44.3 mmol) and $Pd(PPh_3)_4$ (0.51 g, 0.443 mmol) were suspended in a degassed mixture of 1,4-dioxane (45 mL) and water (15 mL) before being heated to reflux under nitrogen atmosphere for 72 hours. After cooling to room temperature, the suspension was concentrated under reduced pressure to a thick oil. The mixture was then diluted with dichloromethane and washed with brine and water. The combined organic layers were dried over anhydrous $MgSO_4$, filtered, and the solvent was removed under reduced pressure. The crude brown product was purified by column chromatography over silica gel (100% ethyl acetate to 5% triethylamine in ethyl acetate gradient) to yield a microcrystalline yellow solid (0.197 g, 20 %). ^{1}H NMR (500 MHz, $CDCl_3$) δ = 8.67 (broad d, 4H, $H^{3'}$), 7.61 (d, $J_{H2'-H3'}$ = 6.1 Hz, 4H, $H^{2'}$), 7.02 (s, 2H, H^{2}); $^{13}C\{^{1}H\}$ NMR (126 MHz, $CDCl_3$) δ = 152.5 (C^{1}), 150.4 ($C^{3'}$), 137.0 ($C^{1'}$), 118.2 ($C^{2'}$), 111.4 (C^{2}). *m/z* (HRMS) *(CI)* 223.0863 $(M+H)^{+}$. $C_{14}H_{11}N_2S$ calc. 223.0871.

1.2 Synthesis of 2,5-di(pyridin-4-yl)thiophene (2)

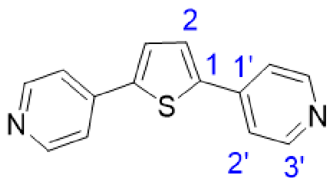

A suspension of 4-pyridinylboronic acid (1.27 g, 10.32 mmol), 2,5-dibromothiophene (0.5 g, 2.06 mmol), $Pd(PPh_3)_4$ (0.238 g, 0.21 mmol) and potassium carbonate (2.9 g, 20.6 mmol) in degassed dioxane/water (15 mL / 5 mL) was refluxed for 48 hours under N_2 atmosphere. After cooling down to room temperature, the solvent was evaporated under reduced pressure and diluted with water. The resultant mixture was extracted with dichloromethane and the organic layer was dried over anhydrous magnesium sulfate and evaporated under reduced pressure. Purification by column chromatography over silica (100% ethyl acetate to 5% triethylamine in ethyl acetate gradient, Rf = 0.3), followed by recrystallisation from ethyl acetate (-18 °C) afforded the title compound as bright yellow solid (0.272 g, 55 %). ^{1}H NMR (500 MHz, $CDCl_3$) δ = 8.64 (broad d, 4H, $H^{3'}$), 7.53 (s, 2H, H^2), 7.51 (d, $J_{H2'-H3'}$ = 6.0 Hz, 4H, $H^{2'}$). ^{13}C{^{1}H} NMR (126 MHz, $CDCl_3$) δ = 150.7 ($C^{3'}$), 142.8 (C^1), 140.9 ($C^{1'}$), 126.7 (C^2), 119.9 ($C^{2'}$). *m/z* (HRMS) *(CI)* 239.0652 $(M+H)^+$. $C_{14}H_{11}N_2S$ calc. 239.0643.

1.3 Synthesis of 2,5-di(pyridin-4-yl)selenophene (3)

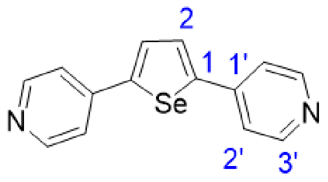

2,5-Dibromoselenophene (2.22 g, 7.69 mmol), 4-pyridylboronic acid (4.71 g, 38.40 mmol), potassium carbonate (10.7 g, 77.4 mmol) and $Pd(PPh_3)_4$ (0.89 g, 0.767 mmol) were suspended in a degassed mixture of 1,4-dioxane (45 mL) and water (15 mL) before being heated under nitrogen at reflux (120 °C) for 65 hrs. The suspension was then allowed to return to room temperature, the solvent was removed *in vacuo*, the residue was dissolved in dichloromethane, and water was added. The product was then extracted with dichloromethane (3 × 30 mL), the combined organic layers were washed with water and brine, dried over anhydrous magnesium sulfate, filtered and the solvent removed *in vacuo*. The crude brown product was purified by column chromatography over silica gel (100% ethyl acetate to 5% triethylamine in ethyl acetate gradient) to afford a yellow powder. This solid was then further purified by two recrystallizations by slow diffusion of pentane to dichloromethane solution of the compound to yield the product as a crystalline yellow solid (91.4 mg, 4 %). ^{1}H NMR (500 MHz, $CDCl_3$) δ = 8.63 (br. d, 4H, $H^{3'}$), 7.73 (s, 2H, H^2), 7.47 (d, $J_{H2'-H3'}$ = 5.7 Hz, 4H, $H^{2'}$). ^{13}C{^{1}H} NMR (126 MHz, $CDCl_3$) δ = 150.3 ($C^{3'}$), 149.1 (C^1), 143.3 ($C^{1'}$), 129.3 (C^2), 120.5 ($C^{2'}$). *m/z* (HRMS) *(CI)* 287.0092 $(M+H)^+$. $C_{14}H_{11}N_2Se$ calc. 287.0087.

1.4 Synthesis of 2,5-di(pyridin-4-yl)tellurophene (4)

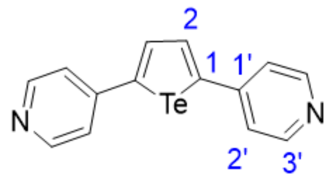

To a degassed solution of ethanol/water (160/6 ml), tellurium powder (1.03 g, 8.07 mmol) and sodium borohydride (1.22 g, 32.35 mmol) were added, and the mixture was heated reflux for 1 hour and 15 minutes, under nitrogen atmosphere. A purple-coloured suspension appeared, and the reaction temperature was reduced to 80 °C. A solution of 1,4-di(pyridin-4-yl)buta-1,3-diyne[2] (551 mg, 2.69 mmol) in degassed ethanol (160 mL) was transferred by cannula to the reaction flask, and the reaction mixture was stirred at 80 °C overnight. After cooling to room temperature, a stream of air was blown over the reaction mixture for 15 minutes. The black precipitate that was formed was removed by filtration through celite and the celite was then washed with dichloromethane until the filtrate no longer contained product as evidenced by spotting on a TLC plate. The dichloromethane filtrate was then washed with brine, dried over anhydrous $MgSO_4$, and filtered. The solvent was removed by rotary evaporator and the crude solid was purified by column chromatography over silica gel (1:1 ethyl acetate/hexane) and then recrystallized from dichloromethane/ethanol to afford **4** as a crystalline yellow solid (87 mg, 10 %). ^{1}H NMR (500 MHz, $CDCl_3$) δ = 8.60 (dd, *J* = 4.6, 1.5 Hz, 4H, $H^{3'}$), 8.10 (s, 2H, H^2), 7.36 (dd, *J* = 4.6, 1.5 Hz, 4H, $H^{2'}$). ^{13}C{^{1}H} NMR (126 MHz, $CDCl_3$) δ = 150.7($C^{3'}$), 147.9 (C^1), 146.8 ($C^{1'}$), 136.6 (C^2), 121.2 ($C^{2'}$). *m/z* (HRMS) *(CI)* 336.9971 $(M+H)^+$. $C_{14}H_{11}N_2Te$ calc. 336.9984.

1.5 Synthesis of 2,5-dibromo-1-methylpyrrole

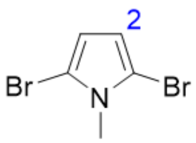

Adapted from Faigl *et al.*[1] In an oven-dried Schlenk flask evacuated and backfilled with N_2, a dimethylformamide (4.0 mL) solution of N-bromosuccinimide (656 mg, 3.69 mmol) was slowly added to a dimethylformamide (6.0 mL) solution of 1-methylpyrrole (150.0 mg, 1.85 mmol) at 0 °C and the mixture was stirred at this temperature for 2 hours. The reaction mixture was then extracted with hexanes (3 × 30 mL), the combined organic layers were washed with water and brine, dried over anhydrous magnesium sulfate, filtered and the solvent removed *in vacuo*. The obtained 2,5-dibromo-1-methylpyrrole was used as the starting material for the next reaction without further purification. ^{1}H NMR (500 MHz, $CDCl_3$) δ 6.20 (s, 2H, H^2), 3.61 (s, 3H, Me).

1.6 Synthesis of 1-Methyl-2,5-bis(4-pyridyl)pyrrole (11)

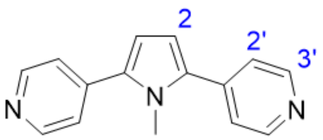

Synthesised following a modified published procedure.[4] A mixture of crude 2,5-dibromo-1-methylpyrrole (1.85 mmol), 4-(4,4,5,5-tetramethyl-1,3,2-dioxaborolan-2-yl)pyridine (832 mg, 4.06 mmol), was suspended in a mixture of 1 M aqueous solution (14 mL) of sodium carbonate (1.56 g, 14.76 mmol) and dimethoxyethane (14 mL) to a Schlenk flask and degassed by bubbling N_2 through, for 30 mins. $Pd(PPh_3)_4$ (213 mg, 0.18 mmol) was added under a flow of nitrogen and the reaction mixture was heated to reflux overnight under N_2 atmosphere (85°C, 20 h). Water was then added to the crude mixture, and the product was extracted into dichloromethane (3 × 30 mL). The combined organic phase was then dried over anhydrous $MgSO_4$, filtered, and the solvent was removed under reduced pressure. The residue was purified by silica gel column chromatography eluting with CH_2Cl_2:methanol (95:5 in volume) to give the title compound as a pale yellow solid. The product was further recrystallized from chloroform to afford pale yellow crystals (145 mg, 33 %). The experimental data obtained are in good agreement with the literature. ^{1}H NMR (500 MHz, $CDCl_3$) δ 8.68 (d, J = 5.8 Hz, 4H, $H^{3'}$), 7.42 (d, J = 6.0 Hz, 4H, $H^{2'}$), 6.56 (s, 2H, H^{2}), 3.76 (s, 3H, Me); ^{13}C{^{1}H} NMR (126 MHz, $CDCl_3$) δ 149.9, 140.5, 136.5, 122.7, 112.0, 35.2. *m/z* (HRMS) *(CI)* 236.1175 $(M+H)^+$. $C_{15}H_{14}N3$ calc. 236.1188.

1.7 NMR Spectra

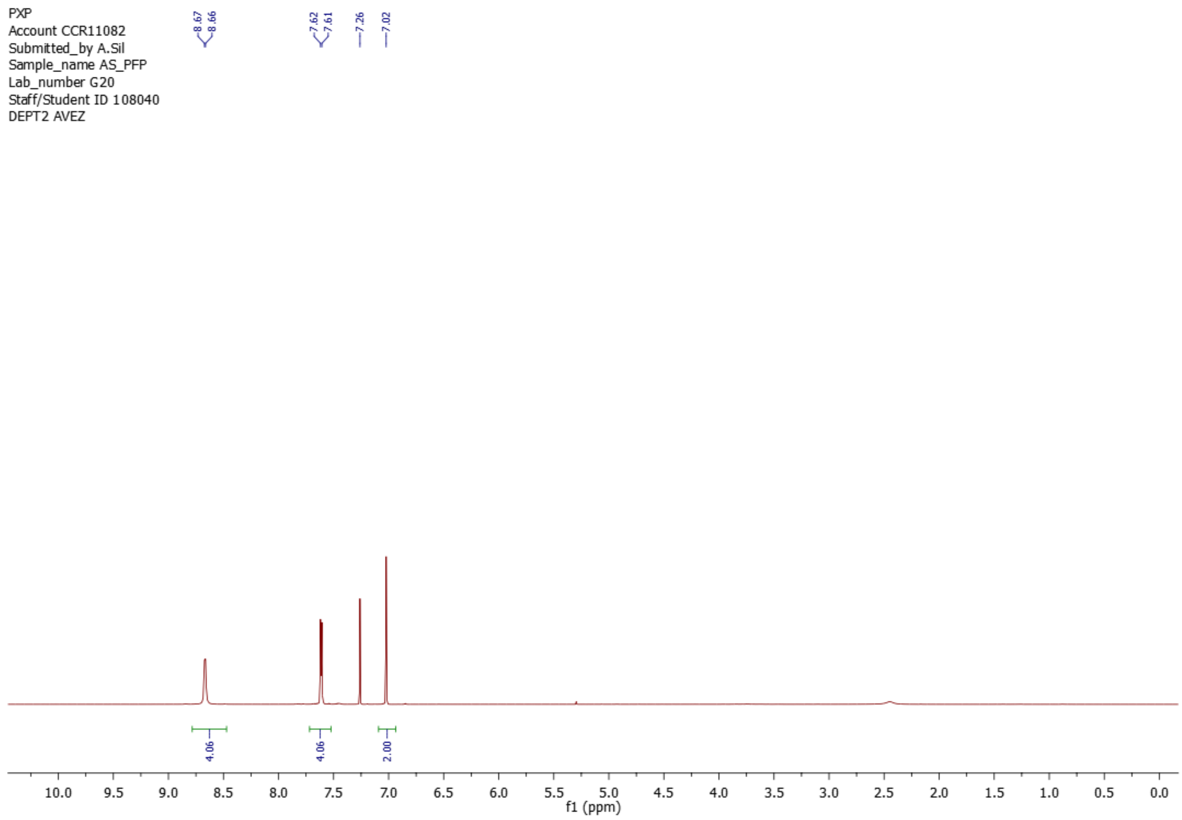

Figure S1: ^{1}H NMR (500 MHz, $CDCl_3$) spectrum of **1**.

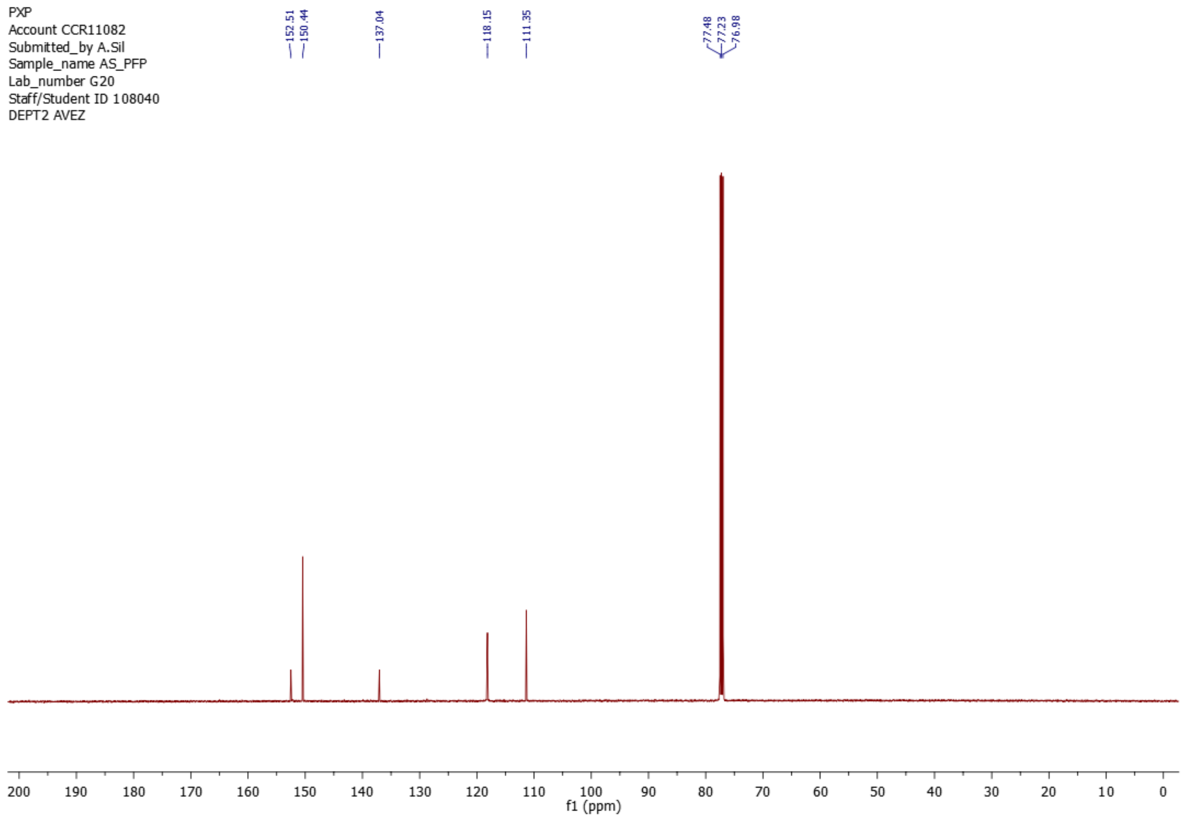

Figure S2: $^{13}C\{^{1}H\}$ NMR (126 MHz, $CDCl_3$) spectrum of **1**.

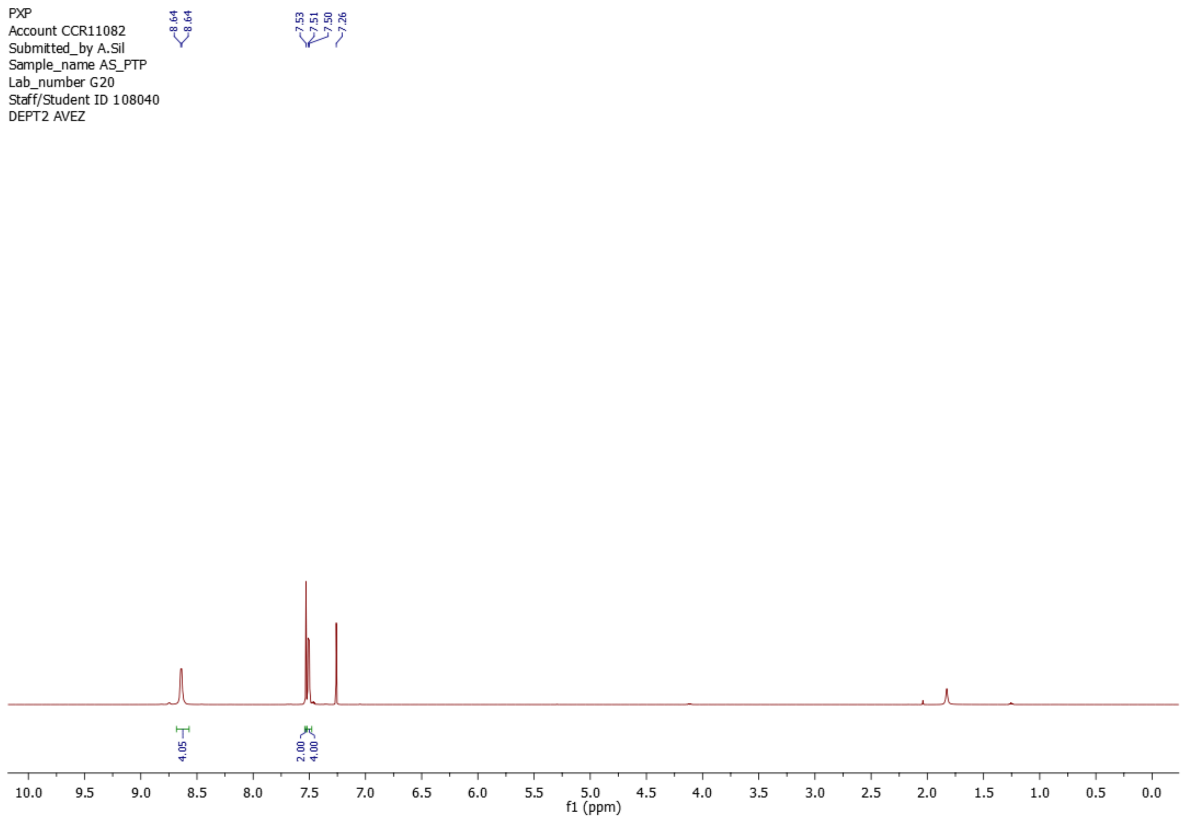

Figure S3: ^{1}H NMR (500 MHz, $CDCl_3$) spectrum of **2**.

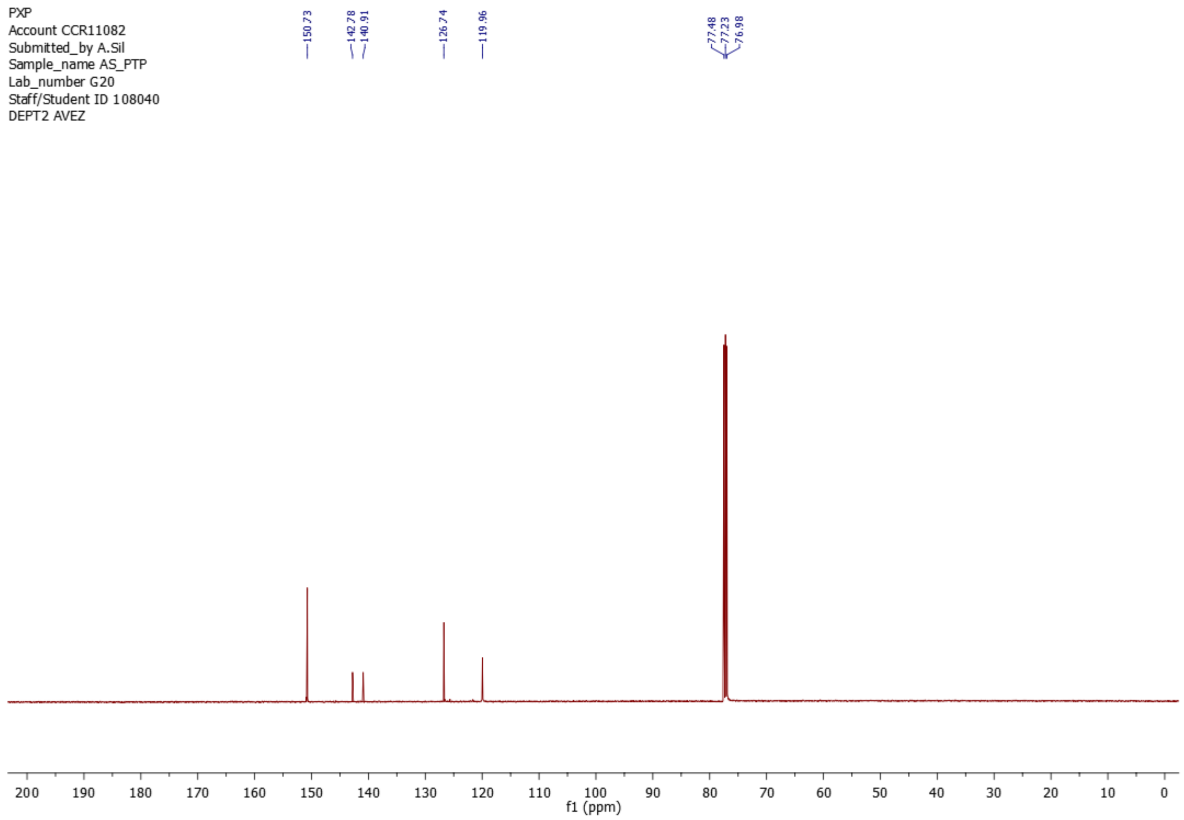

Figure S4: $^{13}C\{^{1}H\}$ NMR (126 MHz, $CDCl_3$) spectrum of **2**.

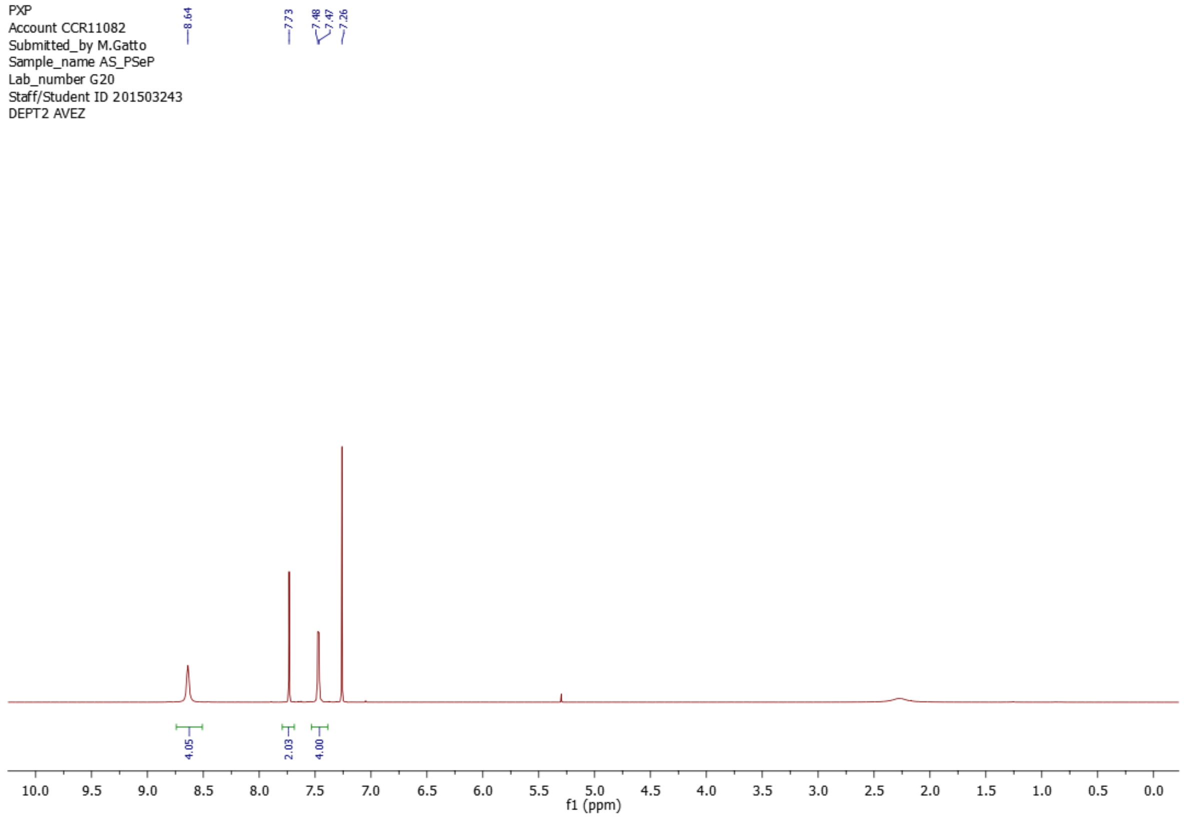

Figure S5: ^{1}H NMR (500 MHz, $CDCl_3$) spectrum of **3**.

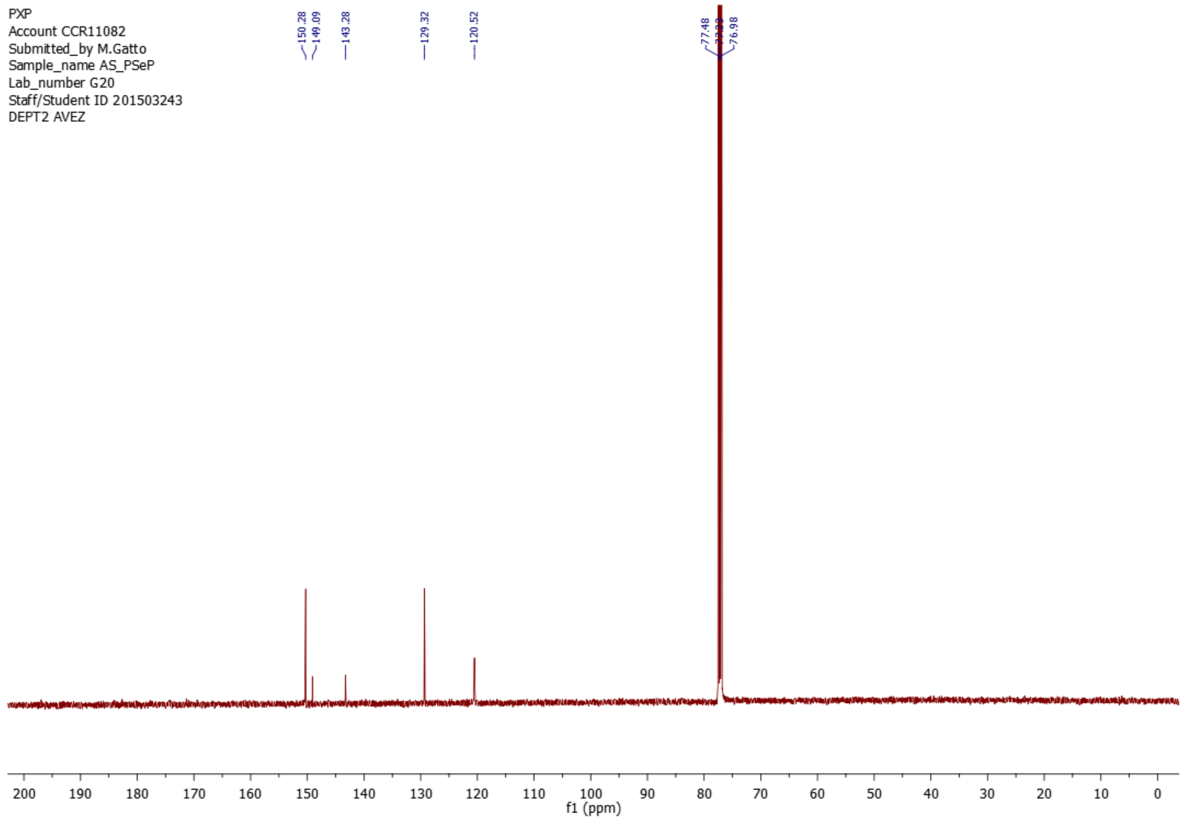

Figure S6: $^{13}C\{^{1}H\}$ NMR (126 MHz, $CDCl_3$) spectrum of **3**.

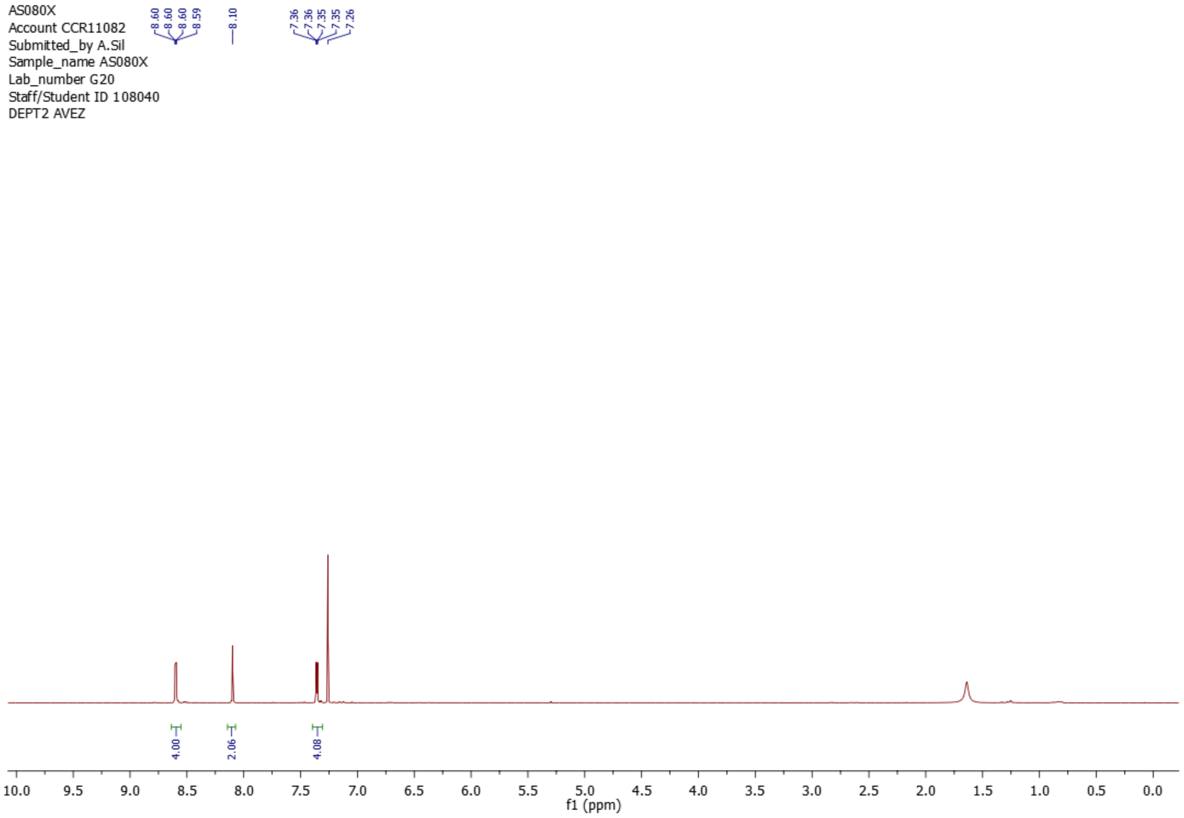

Figure S7: ^{1}H NMR (500 MHz, $CDCl_3$) spectrum of **4**

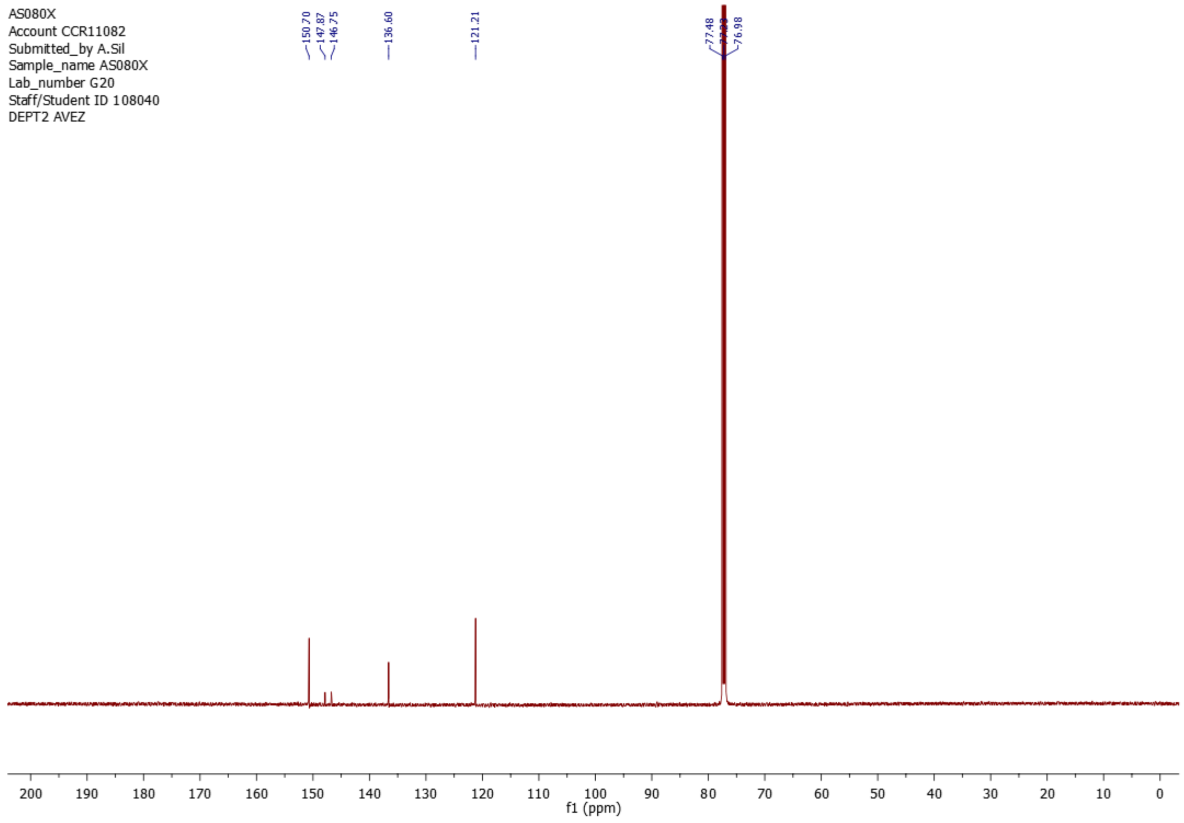

Figure S8: $^{13}C\{^{1}H\}$ NMR (126 MHz, $CDCl_3$) spectrum of **4**.

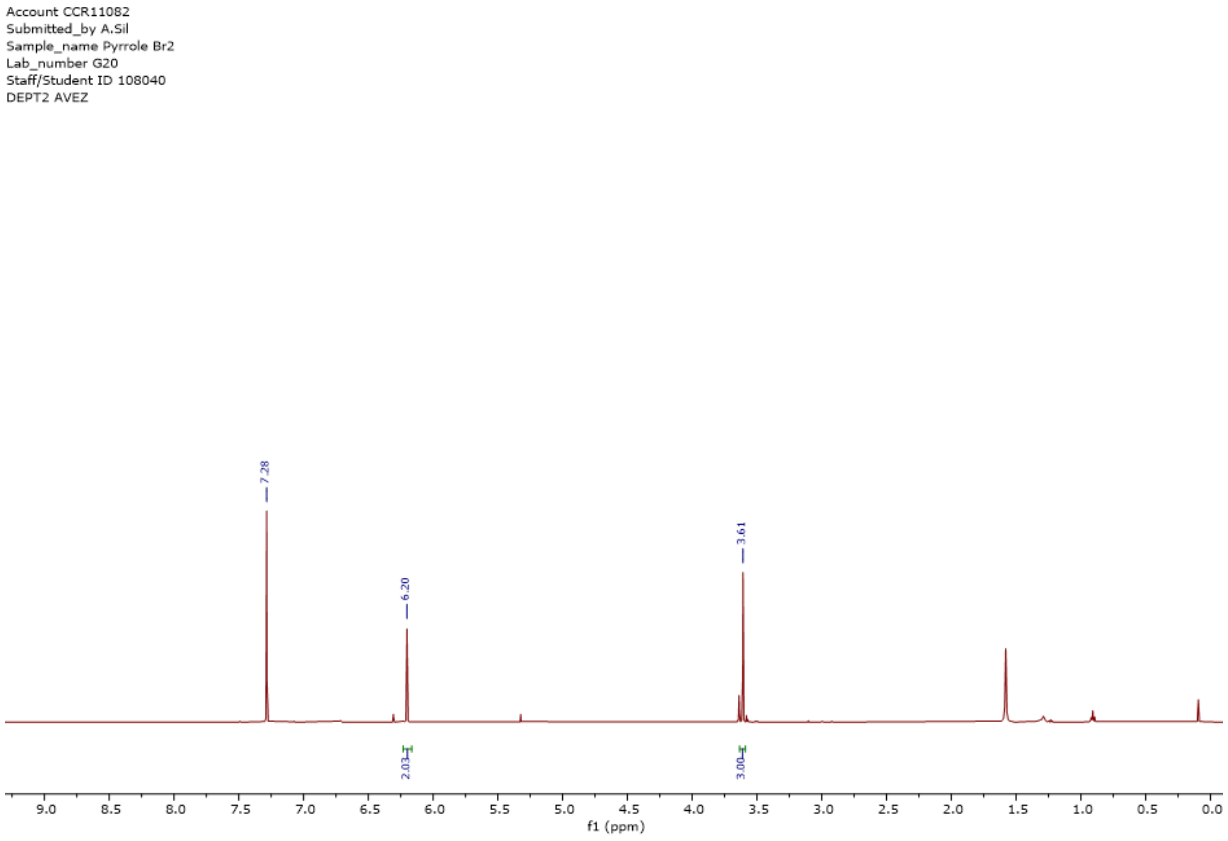

Figure S9: ^{1}H NMR (500 MHz, $CDCl_3$) spectrum of 2,5-dibromo-1-methylpyrrole.

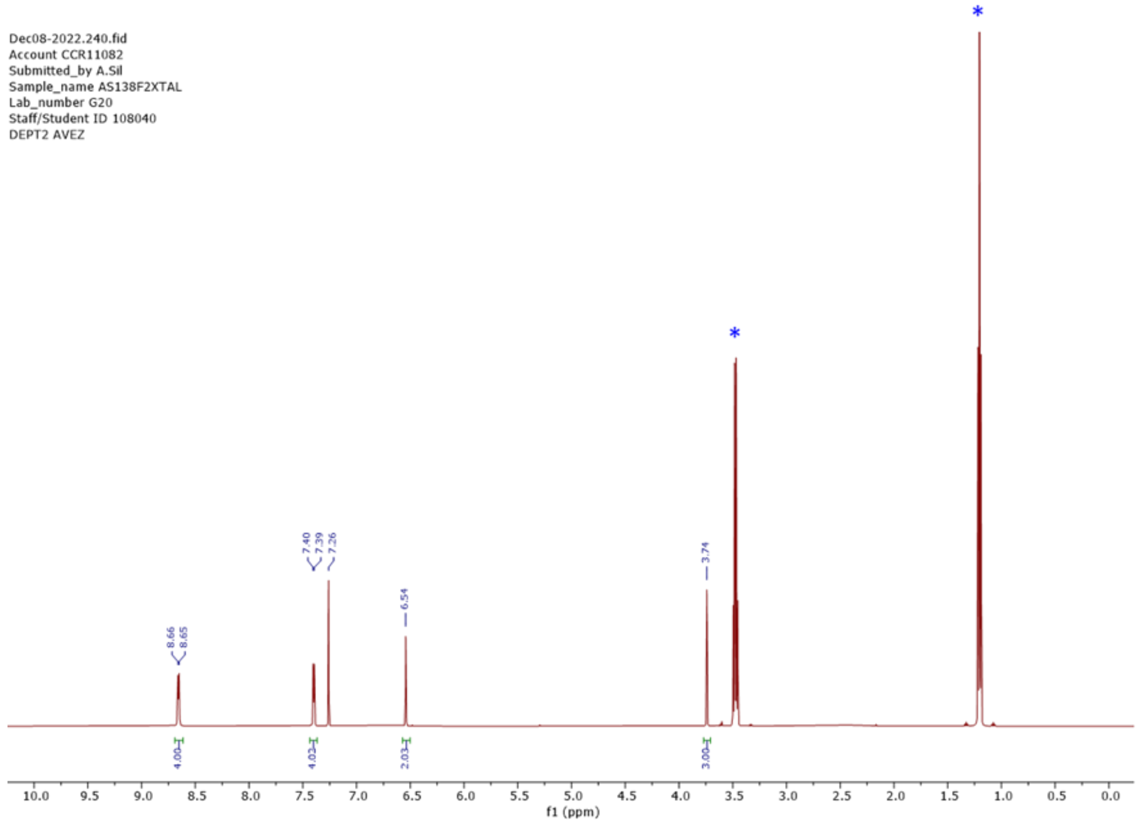

Figure S10: ^{1}H NMR (500 MHz, $CDCl_3$) spectrum of **11** (* = diethyl ether).

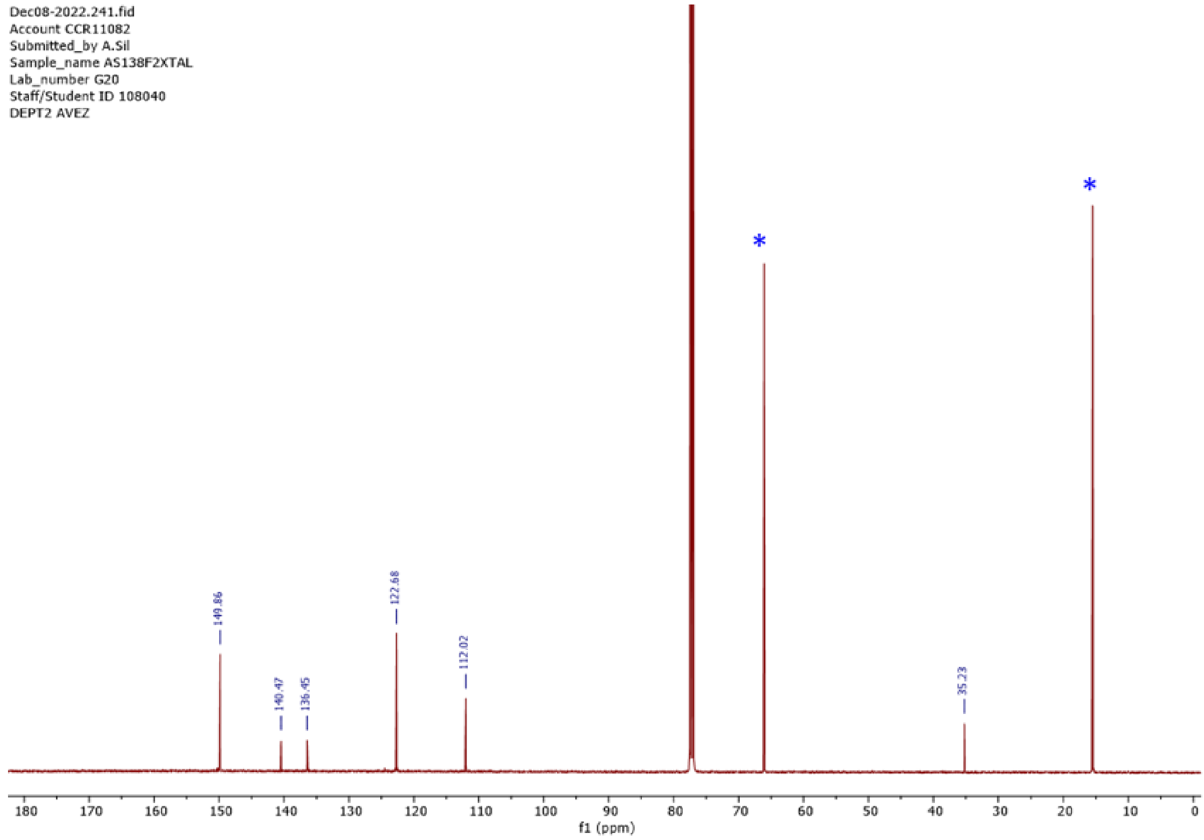

Figure S11: $^{13}C\{^{1}H\}$ NMR (126 MHz, $CDCl_3$) spectrum of **11** (* = residual diethyl ether).

1.8 Crystallographic Characterisation of 4

Single crystals of $C_{14}H_{10}N_2Te$, **4**, were submitted for single crystal X-ray determination. A suitable crystal was selected and mounted on a MiTeGen tip using Parabol oil and placed on a Bruker D8 Venture diffractometer. The crystal was kept at 200.0 K during data collection. Using Olex2[5], the structure was solved with the XT[6] structure solution program using Intrinsic Phasing and refined with the XL[7] refinement package using Least Squares minimisation.

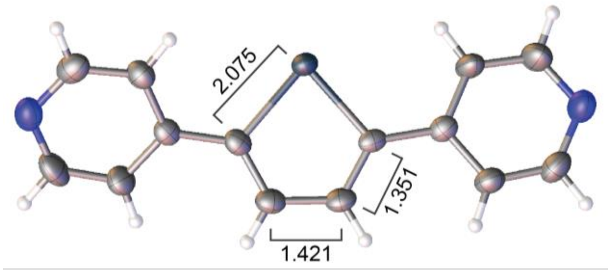

Figure S12: Crystallographic structure of **4**, with bond lengths in the tellurophenyl moiety in Å.

Table S1 Crystal data and structure refinement for **4**.

Identification code	AS080_final
Empirical formula	$C_{14}H_{10}N_2Te$
Formula weight	333.84
Temperature/K	200.0
Crystal system	tetragonal
Space group	$P4_32_12$
a/Å	8.6780(6)
b/Å	8.6780(6)
c/Å	31.724(2)
α/°	90
β/°	90
γ/°	90
Volume/Å^3	2389.1(4)
Z	8
ρ_{calc}g/cm^3	1.856
μ/mm^{-1}	2.467
F(000)	1280.0
Crystal size/mm^3	0.25 × 0.2 × 0.05
Radiation	MoKα (λ = 0.71073)
2Θ range for data collection/°	4.866 to 52.746
Index ranges	$-7 \leq h \leq 10$, $-10 \leq k \leq 10$, $-39 \leq l \leq 37$
Reflections collected	8580
Independent reflections	2436 [R_{int} = 0.0299, R_{sigma} = 0.0316]
Data/restraints/parameters	2436/0/155
Goodness-of-fit on F^2	1.059
Final R indexes [I>=2σ (I)]	R_1 = 0.0226, wR_2 = 0.0401
Final R indexes [all data]	R_1 = 0.0276, wR_2 = 0.0411
Largest diff. peak/hole / e Å^{-3}	0.23/-0.27
Flack parameter	0.019(18)

Table S2 Fractional Atomic Coordinates (×10^4) and Equivalent Isotropic Displacement Parameters (Å^2×10^3) for **4**. U_{eq} is defined as 1/3 of of the trace of the orthogonalised U_{IJ} tensor.

Atom	*x*	*y*	*z*	U(eq)
Te1	8571.4(3)	7383.8(3)	6088.5(2)	34.09(11)
C4	7246(4)	8152(4)	5586.1(11)	26.0(9)
N1	7560(4)	1540(4)	6846.3(11)	43.9(8)
C5	7313(4)	4144(4)	6311.2(11)	28.9(8)
N2	7995(4)	12528(5)	4986.3(11)	45.8(9)
C3	6166(5)	7089(5)	5488.8(12)	33.1(10)
C6	8047(5)	4218(5)	6699.2(13)	35.5(10)
C9	6701(5)	2705(5)	6200.6(13)	40.0(10)
C10	7507(5)	9642(5)	5383.2(11)	29.9(9)
C2	6127(5)	5679(5)	5716.1(12)	33.3(10)
C14	8764(5)	10556(5)	5471.7(14)	43.9(12)
C1	7165(4)	5468(4)	6033.4(12)	29.9(9)
C7	8130(5)	2916(5)	6949.5(14)	43.5(12)
C11	6496(5)	10232(5)	5079.6(12)	38.1(10)
C8	6855(5)	1478(5)	6472.9(16)	48.5(12)
C12	6792(5)	11631(6)	4896.8(13)	43.7(12)
C13	8959(5)	11943(6)	5275.6(15)	51.1(13)

Table S3 Anisotropic Displacement Parameters (Å^2×10^3) for **4**. The Anisotropic displacement factor exponent takes the form: $-2\pi^2[h^2a^{*2}U_{11}+2hka^*b^*U_{12}+\ldots]$.

Atom	U_{11}	U_{22}	U_{33}	U_{23}	U_{13}	U_{12}
Te1	30.84(16)	32.67(17)	38.76(16)	3.38(14)	-11.70(12)	-2.46(13)
C4	21(2)	33(2)	24.4(18)	-6.4(15)	0.0(16)	6.5(17)
N1	38(2)	41(2)	54(2)	10.8(19)	5(2)	1(2)
C5	25(2)	27.2(19)	35(2)	-3.9(16)	5.4(19)	3.3(17)
N2	40(2)	49(2)	48(2)	16(2)	-0.1(17)	-1(2)
C3	31(2)	43(3)	25.4(19)	-4.7(18)	-3.8(18)	-0.2(19)
C6	34(2)	35(2)	38(2)	1(2)	4.6(19)	-4.9(18)
C9	45(2)	31(2)	44(2)	-4(2)	-4.3(19)	1(2)
C10	23(2)	39(2)	27.4(19)	1.2(17)	3.7(19)	3(2)
C2	30(2)	35(2)	34(2)	-8.4(19)	-1.7(19)	0.1(18)
C14	29(2)	53(3)	49(3)	17(2)	-12(2)	-8(2)
C1	28(2)	26.8(19)	35(2)	-5.5(18)	3.6(19)	0.5(17)
C7	31(2)	59(3)	40(2)	8(2)	2.1(19)	0(2)
C11	31(2)	46(3)	37(2)	3(2)	-6(2)	0(2)
C8	47(3)	27(2)	71(3)	-8(3)	4(2)	-4(2)
C12	40(3)	52(3)	39(2)	9(2)	-6(2)	8(2)
C13	36(3)	57(3)	60(3)	17(3)	-8(2)	-14(2)

Table S4 Bond Lengths for **4**.

Atom	Atom	Length/Å	Atom	Atom	Length/Å
Te1	C4	2.075(4)	N2	C13	1.342(6)
Te1	C1	2.070(4)	C3	C2	1.421(6)
C4	C3	1.351(5)	C6	C7	1.383(6)
C4	C10	1.462(5)	C9	C8	1.377(6)
N1	C7	1.334(5)	C10	C14	1.378(6)
N1	C8	1.334(6)	C10	C11	1.400(5)
C5	C6	1.388(5)	C2	C1	1.363(5)
C5	C9	1.402(5)	C14	C13	1.365(6)
C5	C1	1.454(5)	C11	C12	1.369(6)
N2	C12	1.333(5)			

Table S5 Bond Angles for **4**.

Atom	Atom	Atom	Angle/˚	Atom	Atom	Atom	Angle/˚
C1	Te1	C4	82.31(15)	C14	C10	C11	115.2(4)
C3	C4	Te1	109.9(3)	C11	C10	C4	122.0(4)
C3	C4	C10	127.6(4)	C1	C2	C3	118.3(4)
C10	C4	Te1	122.4(3)	C13	C14	C10	120.9(4)
C7	N1	C8	115.1(4)	C5	C1	Te1	122.1(3)
C6	C5	C9	115.9(4)	C2	C1	Te1	110.1(3)
C6	C5	C1	122.8(4)	C2	C1	C5	127.7(4)
C9	C5	C1	121.3(3)	N1	C7	C6	124.8(4)
C12	N2	C13	114.4(4)	C12	C11	C10	119.9(4)
C4	C3	C2	119.2(4)	N1	C8	C9	124.8(4)
C7	C6	C5	119.7(4)	N2	C12	C11	125.1(4)
C8	C9	C5	119.7(4)	N2	C13	C14	124.6(4)
C14	C10	C4	122.8(4)				

Table S6 Hydrogen Atom Coordinates (Å×10^4) and Isotropic Displacement Parameters (Å^2×10^3) for **4**.

Atom	*x*	*y*	*z*	U(eq)
H3	5440.05	7282.69	5270.82	40
H6	8490.77	5159.42	6792.48	43
H9	6183.05	2575.71	5939.12	48
H2	5395.35	4902.48	5648.68	40
H14	9504.6	10217	5671.78	53
H7	8631.06	3009.21	7214.55	52
H11	5606.57	9663.29	5000.48	46
H8	6428.23	518.31	6388.73	58
H12	6084.68	11989.14	4690.29	52
H13	9839.43	12535	5349.14	61

2. STMBJ Methods and Additional Data

We used a modified commercial STM (Keysight Technologies 5500 SPM) in our measurements. The system is equipped with a custom-made 4-channel preamplifier based on the design by Meszaros *et al.*[8] with a National Instruments NI9215 USB data acquisition board (16-bit, 10 KSa/s). Au substrates for *STMBJ* measurements were fabricated by e-beam evaporation (Korvustech HEX Tau4) of ~200 nm Au (99.99+%, Advent Research Materials) on freshly cleaved muscovite mica (Agar Scientific). Substrates were briefly annealed with a butane torch before use. Tips were cut from a spool of Au wire (99.998+%, ThermoFisher PREMION, 0.25 mm Ø). Measurements were performed in a mixture of mesitylene and THF, 4:1 v:v, with the target molecule in a 1 mM concentration, at room temperature. All data was acquired and processed with custom software written in Python. Data was continuously acquired as the tip was moved into and out of contact with the substrate at a rate of 12 nm/s. If the system repeatedly failed to engage with the substrate at conductance $G \gg G_0$ or conductance did not decay to the noise level ($10^{-5.3}$ G_0 at 100 mV bias) within 6 nm of tip withdrawal, data acquisition was stopped, and the tip moved to a different area on the substrate using the *XY* piezoelectric transducers of the STM. Data collection was then restarted. The measured conductance traces were aligned to the G_0 rupture and compiled into log-binned conductance histograms and 2D density maps with no further data selection. All histograms in the SI are presented with 100 bins per conductance decade and 30 bins per nanometre.

2.1 Histogram and Density Plot for compound 1

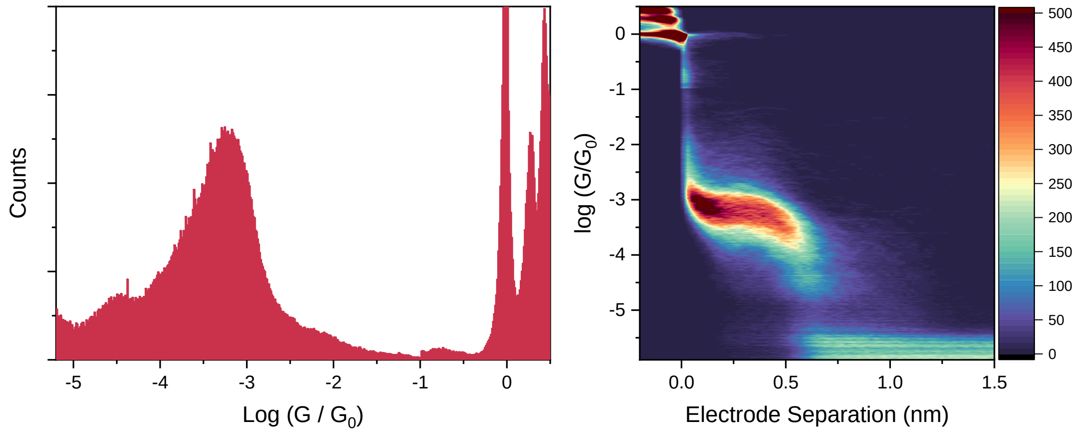

Figure S13: Histogram (left) and 2D density map (right) for the furan derivative **1**. 100 mV bias, 1 mM mesitylene:THF 8:2 v:v solution, 4258 individual STMBJ traces.

2.2 Histogram and Density Plot for compound 2

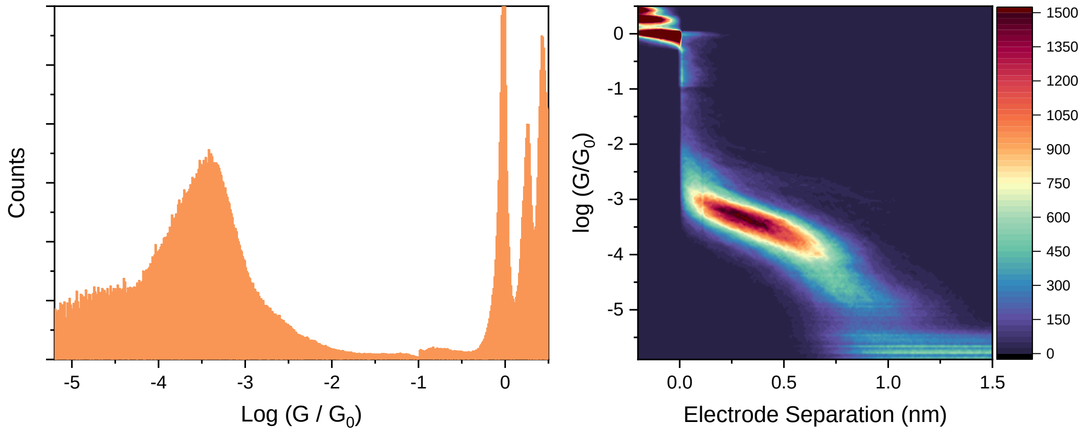

Figure S14: Histogram (left) and 2D density map (right) for the thiophene derivative **2**. 100 mV bias, 1 mM mesitylene:THF 8:2 v:v solution, 4784 individual STMBJ traces.

2.3 Histogram and Density Plot for compound 3

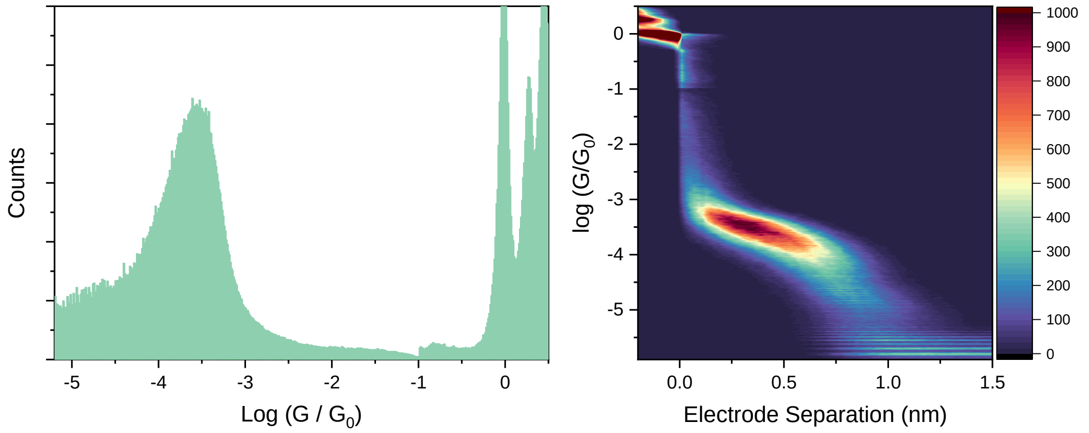

Figure S15: Histogram (left) and 2D density map (right) for the selenophene derivative **3**. 100 mV bias, 1 mM mesitylene:THF 8:2 v:v solution, 5188 individual STMBJ traces.

2.4 Histogram and Density Plot for compound 4

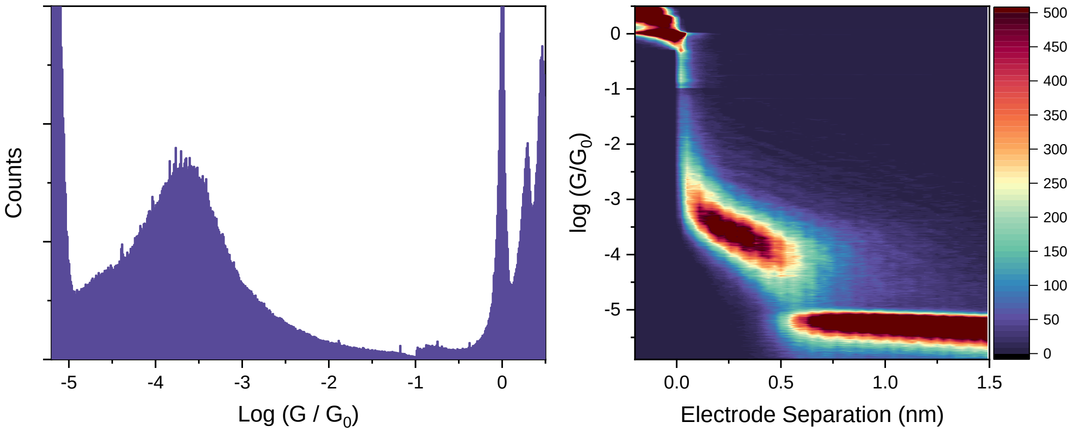

Figure S16: Histogram (left) and 2D density map (right) for the tellurophene derivative **4**. 100 mV bias, 1 mM mesitylene:THF 8:2 v:v solution, 5454 individual STMBJ traces.

2.5 Histogram and Density Plot for compound 11

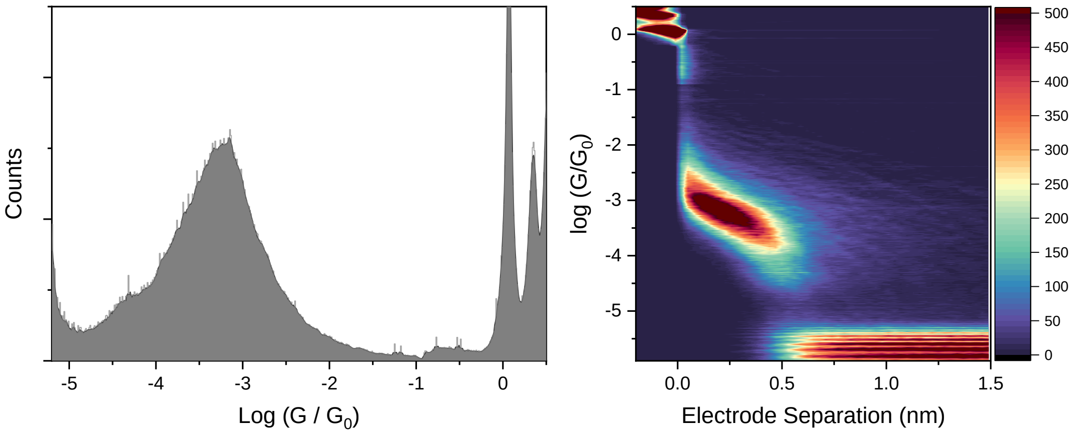

Figure S17: Histogram (left) and 2D density map (right) for the pyrrole derivative **5**. 100 mV bias, 1 mM mesitylene:THF 8:2 v:v solution, 4889 individual STMBJ traces.

3. NMR – Conductance Correlation

3.1 Further Details on 1-4

In addition to the plots shown in the main manuscript, we report here the correlation between the $^{13}C\{^{1}H\}$ NMR peak position and single-molecule conductance.

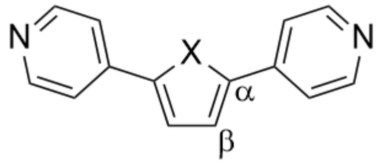

	^{1}H NMR ppm (β)	$^{13}C\{^{1}H\}$ NMR (α)	$^{13}C\{^{1}H\}$ NMR (β)	Log (G / G_0)	Gaussian Fit σ
**1**	7.02	152.5	111.3	-3.25	0.32
**2**	7.53	142.8	126.7	-3.43	0.35
**3**	7.73	149.1	129.3	-3.52	0.42
**4**	8.1	147.9	136.6	-3.68	0.51

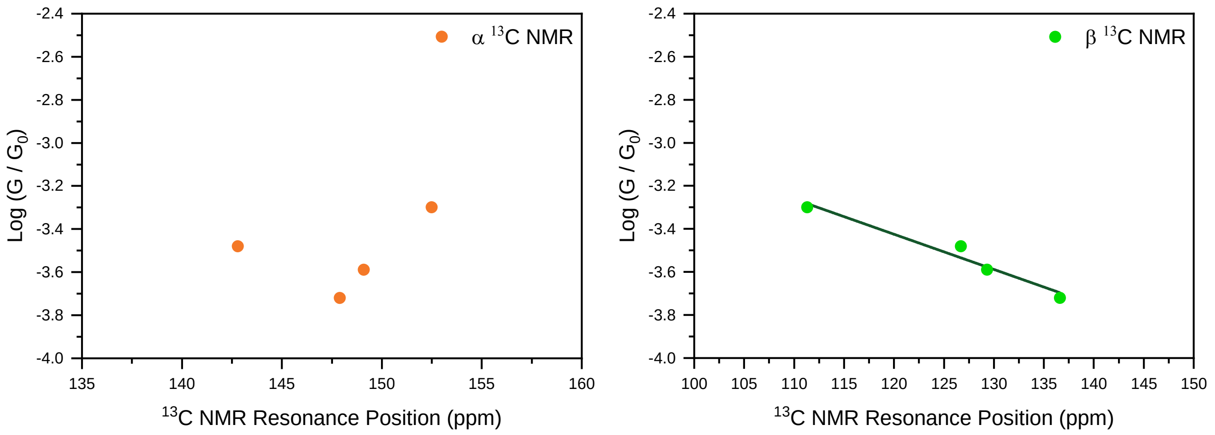

Figure S18: NMR signals for compounds **1-**4 and correlation between $^{13}C\{^{1}H\}$ NMR signals and molecular conductance.

As can be observed in Figure S18, there is no evident correlation between the $^{13}C\{^{1}H\}$ signal for the nuclei in position α respective to the heteroatom, but the position of the resonance arising from the nuclei in β follows a dependence similar to that observed for the β protons.

3.2 NMR DFT calculations

We performed DFT calculations using the Wavefun® implementation of the Q-CHEM package, using B3LYP functionals and the 6-31G* basis set for **1**, and the 6-311+G(2df,2p) for increased accuracy in **2** and **3**. We tested Dunning basis sets (cc-pV) for **4** but despite our best efforts, calculations failed to converge. NMR chemical shielding tensors were

calculated using gauge-including atomic orbitals (GIAOs), and the chemical shift was obtained referenced to tetramethylsilane (calculated again at the B3LYP/6-31G* level of theory) as $\delta = \Delta\sigma$, with the calculated $\sigma_{TMS} = 32.1884$.

The results of our calculations for the β protons (^{1}H NMR) are shown in the table below, with the isotropic shielding tensor calculated using the IUPAC convention and the anisotropic contribution calculated using within the Haberlen convention. Values are rounded to the 4th decimal place.

	**^{1}H NMR exp. δ ppm**	**^{1}H NMR DFT δ ppm**	**Isotropic Shielding Tensor (IUPAC) σ ppm**	**Anisotropy (Haeberlen)**
**1**	7.02	6.83	25.3546	-6.1835
**2**	7.53	7.34	24.8429	-7.7614
**3**	7.73	7.43	24.7545	-7.9624

As discussed in the main paper, DFT simulations follow the experimentally observed trend, with the protons in β respective to the heteroatom of the chalcogenophene being increasingly deshielded, in the **1** → **3** series, while the degree of anisotropy increases.

3.6 Principal Components

We report here the DFT Data as the raw tensor matrix for the proton used in the main text for correlation. We used MATLAB R2022a with the Tensor Toolbox† and the Navigation Toolbox‡ to calculate its symmetrised decomposition, the rotation matrix used to obtain the principal components, and the Euler angles of rotation (2π). DFT calculations were started with the chalcogen in the origin of the cartesian plane, with the N-N axis pointing in the X direction and the molecule lying flat on the XZ plane.

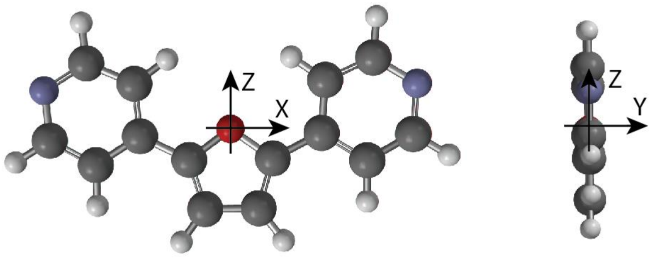

Figure S19: DFT Cartesian Coordinates.

† Available at https://www.tensortoolbox.org/ [Retrieved 23rd February 2024]
‡ Available at https://uk.mathworks.com/products/navigation.html [Retrieved 26th February 2024]

Tensor matrices are reported as: $\begin{matrix} \sigma_{xx} & \sigma_{xy} & \sigma_{xz} \\ \sigma_{yx} & \sigma_{yy} & \sigma_{yz} \\ \sigma_{zx} & \sigma_{zy} & \sigma_{zz} \end{matrix}$

Compound 1:

Raw Tensor: $\begin{matrix} 27.6995 & 0 & -0.9462 \\ 0 & 21.2323 & 0 \\ -0.6574 & 0 & 27.1320 \end{matrix}$

Symmetric Component: $\begin{matrix} 27.6995 & 0 & -0.8018 \\ 0 & 21.2323 & 0 \\ -0.8018 & 0 & 27.1320 \end{matrix}$

Diagonalised: $\begin{matrix} 21.2323 & 0 & 0 \\ 0 & 26.5652 & 0 \\ 0 & 0 & 28.2663 \end{matrix}$

Rotation Matrix: $\begin{matrix} 0 & -0.5772 & -0.8166 \\ 1 & 0 & 0 \\ 0 & -0.8166 & 0.5772 \end{matrix}$

Euler Rotation (XYZ) $\begin{matrix} 0 & -0.9555 & 1.5708 \end{matrix}$

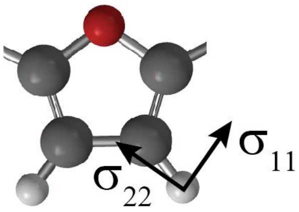

Figure S20: Principal components σ_{11} and σ_{22} for **1**. σ_{33} is the out-of-plane component.

Compound 2:

Raw Tensor: $\begin{matrix} 27.1934 & 0 & -1.7851 \\ 0 & 19.7672 & 0 \\ 0.0802 & 0 & 27.5681 \end{matrix}$

Symmetric Component: $\begin{matrix} 27.1934 & 0 & -0.8525 \\ 0 & 19.7672 & 0 \\ -0.8525 & 0 & 27.5681 \end{matrix}$

Diagonalised: $\begin{matrix} 19.7672 & 0 & 0 \\ 0 & 26.5079 & 0 \\ 0 & 0 & 28.2536 \end{matrix}$

Rotation Matrix: $\begin{matrix} 0 & -0.7793 & -0.6266 \\ 1 & 0 & 0 \\ 0 & -0.6266 & 0.7793 \end{matrix}$

Euler Rotation (XYZ) $\begin{matrix} 0 & -0.6722 & 1.5708 \end{matrix}$

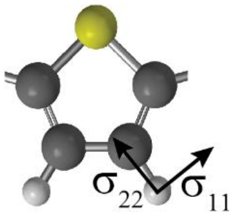

Figure S21: Principal components σ_{11} and σ_{22} for **2**. σ_{33} is the out-of-plane component.

Compound 3:

Raw Tensor:

$$\begin{matrix} 26.8481 & 0 & -1.9563 \\ 0 & 19.4463 & 0 \\ 0.4616 & 0 & 27.9692 \end{matrix}$$

Symmetric Component:

$$\begin{matrix} 26.8481 & 0 & -0.7474 \\ 0 & 19.4463 & 0 \\ -0.7474 & 0 & 27.9692 \end{matrix}$$

Diagonalised:

$$\begin{matrix} 19.4463 & 0 & 0 \\ 0 & 26.4744 & 0 \\ 0 & 0 & 28.3429 \end{matrix}$$

Rotation Matrix:

$$\begin{matrix} 0 & -0.8944 & -0.4472 \\ 1 & 0 & 0 \\ 0 & -0.4472 & 0.8944 \end{matrix}$$

Euler Rotation (XYZ) $\quad 0 \quad -0.4636 \quad 1.5708$

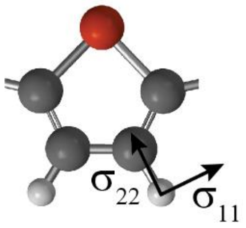

Figure S22: Principal components σ_{11} and σ_{22} for **3**. σ_{33} is the out-of-plane component.

Principal components (using the IUPAC convention) are reported below.

	**Isotropic Shielding Tensor (IUPAC) σ ppm**	**σ_{11} (IUPAC)**	**σ_{22} (IUPAC)**	**σ_{33} (IUPAC)**
**1**	25.3546	28.2663	26.5652	21.2323
**2**	24.8429	28.2536	26.5079	19.7672
**3**	24.7545	28.3429	26.4744	19.4463

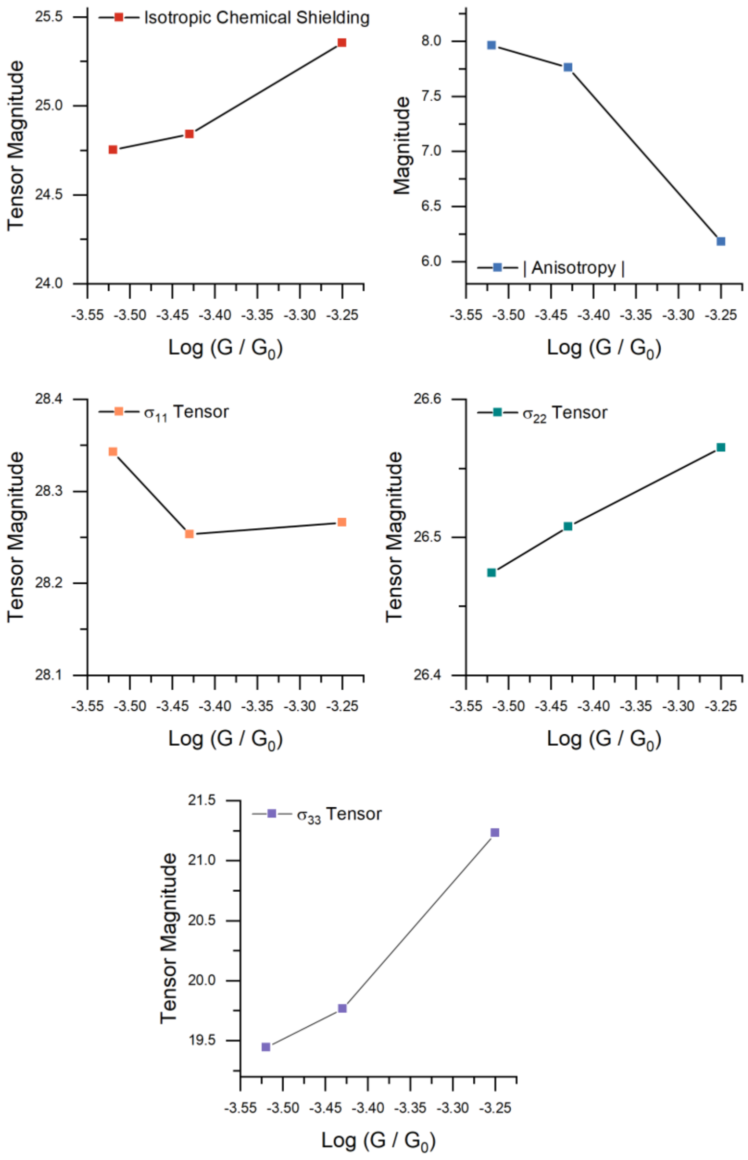

Figure S23: Correlation between NMR parameters and single-molecule conductance for **1-3**.

3.7 Plateau Length Analysis and Correlation at the Most Common Junction Extension

We performed plateau length analysis using an automated algorithm to extract the conductance value at the most common value of junction elongation, which would represent the most idealised configuration of the single-molecule junction – *e.g.* fully stretched between the two Au electrodes.

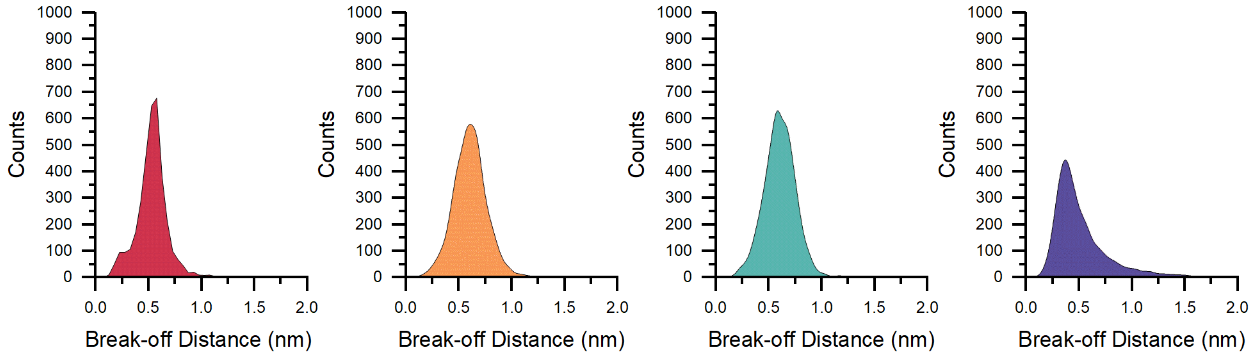

Figure S24: Plateau length distribution for compounds **1-4**

The above distributions of plateau length were fitted to a Gaussian distribution, which are in good agreement with the 2D density maps showed in the previous section of the SI. Slices of the 2D plots at the most probable junction extension were then extracted from the 2D density maps.

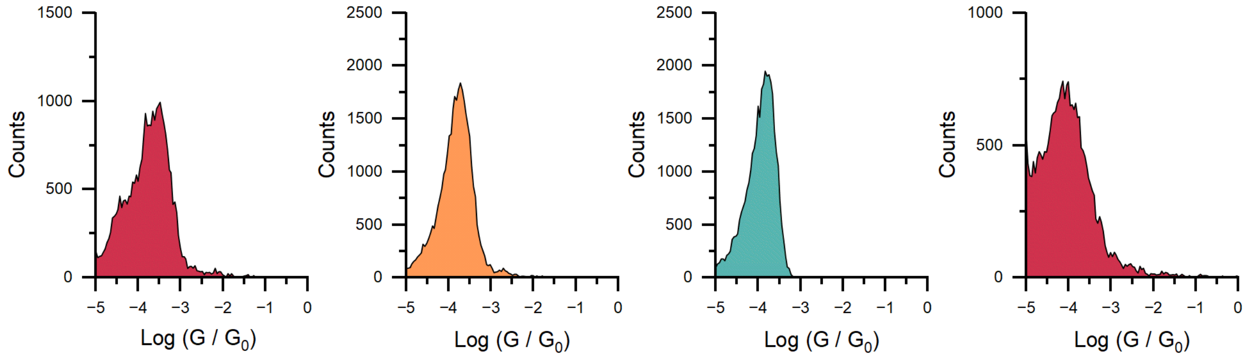

Figure S25: Conductance histograms at the most probable break-off length.

Gaussian fitting of the conductance plots returned the most probable conductance value at the most probable break-off length, thus removing deviating behaviour in the distributions due to different junction evolution profiles across the **1-4** series as the electrodes are pulled apart during a break-junction experiment. Results are reproduced in the table below.

Table S1: Gaussian fittings of the break-off distance distributions. x_c is the centre of the distribution and σ is its standard deviation.

	x_c (nm)	σ	$log\ (G\ /\ G_0)$
**1**	0.54	0.08	-3.61
**2**	0.58	0.11	-3.76
**3**	0.60	0.09	-3.88
**4**	0.48	0.08	-4.11

We recalculated the correlation between NMR chemical shift and single-molecule conductance, and obtained the same result shown in the main text (slope of -0.41 here and -0.39 for the conductance values shown in the main text).

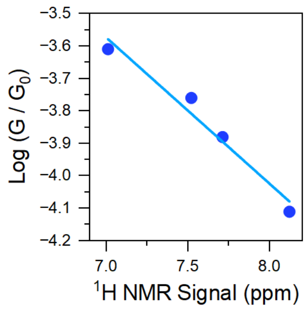

Figure S26: Conductance *vs* 1H NMR resonance position plots for **1-4**, with line fitting as guide to the eye. The conductance value used in this plot is that extracted at the most probable break-off distance, thus representing the most probable, fully-extended junction configuration.

4. Additional Considerations on the Aromaticity of 1-4

The presence of 4-pyridyl substituents on the chalcogenophene could in principle alter their aromaticity. We used two different methods, originally developed by F. Fringuelli[10] and A. Julg,[11] to estimate the degree of aromatic character from the experimentally determined bond length. In the method developed by Fringuelli, the degree of aromaticity is given by the sum of the difference of *bond orders* ΔN (determined using the Gordy[12] equation) of the aromatic ring. In an ideally aromatic structure, such as benzene, this sum $\sum \Delta N$ is equal to zero as all bonds are of the same order. The higher $\sum \Delta N$ is, the less aromatic is a system. In the method developed by Julg, instead, the degree of ring aromaticity is estimated from the deviation of each bond length from their mean value, using the equation

$$J = 1 - \frac{225}{n} \sum_{i=1}^{n} \left(1 - {d_n}/{\bar{d}}\right)^2 \qquad Eq. 1$$

In this case, the lower J is, the less aromatic a ring is, and J for benzene is 1. The results for compounds **1** – **4,** along with the crystallographic bond lengths are reported in Table 1.

Table S1: Crystallographic bond lengths and aromaticity parameters. Values for **1**, **2** and **3** extracted from CIFs available in the CCDC database, entry #1556858, #288922 and #1556855, respectively. Values for **4** obtained from the XRD structure shown in Figure S12.

**Compound**	**C-C (Å)**	**C=C (Å)**	**C-X (Å)**	$\sum \Delta N$	J
**1**	1.415	1.353	1.367	1.55	0.89
**2**	1.399	1.355	1.728	1.19	0.94
**3**	1.414	1.361	1.878	1.45	0.92
**4**	1.421	1.351	2.075	1.71	0.87

While there are some minor discrepancies between these two methods, they clearly establish the thiophene derivative **2** as having strong aromatic character, closely followed by the selenophene derivative **3**. The furan and tellurophene derivatives **1** and **4** are much less aromatic. With Fringuelli's method, **4** displays even lower aromaticity than **1** ($\sum \Delta N$ of 1.71 and 1.55, respectively), while the order is reversed following Julg's method (J of 0.87 for **4** and 0.89 for **1**). We can conclude that the presence of the 4-pyridyl anchors does not have a significant effect on the aromaticity order of the four compounds used in this study from that obtained from spectroscopic data and chemical reactivity presented in the main text.

5. Theoretical Methods

5.1 Tight-Binding Calculations

The tight-binding Hamiltonian of each structure discussed in the main text was combined with the GOLLUM[13,14] implementation of the nonequilibrium Green's function method to calculate the phase-coherent, elastic scattering properties of each system consisting of left (source) and right (drain) leads and the scattering region. The transmission coefficient $T(E)$ for electrons of energy E (passing from the source to the drain) is calculated via the relation $T(E) = trace(\Gamma_R(E)G^R(E)\Gamma_L(E)E^{R^\dagger}(E))$. In this expression, $\Gamma_{L,R}(E) = i(\sum_{L,R}(E) - \sum_{L,R}^{\dagger}(E))$ describes the level broadening due to the coupling between left (L) and right (R) electrodes and the central scattering region, $\Sigma_{L,R}$(E) are the retarded self-energies associated with this coupling, and $G_R = (EI - H - \Sigma_L - \Sigma_R)^{-1}$ is the retarded Green's function, where H is the Hamiltonian and I is identity matrix. Using the obtained transmission coefficient $T(E)$, conductance is calculated using the Landauer formula[15] $G = G_0 \int dE\, T(E) \left(-\frac{\partial f}{\partial E}\right)$ where $G_0 = 2e^2/h$ is the conductance quantum.

We show here the individual TB transmission curves used to construct the theoretical conductance histograms show in the main text.

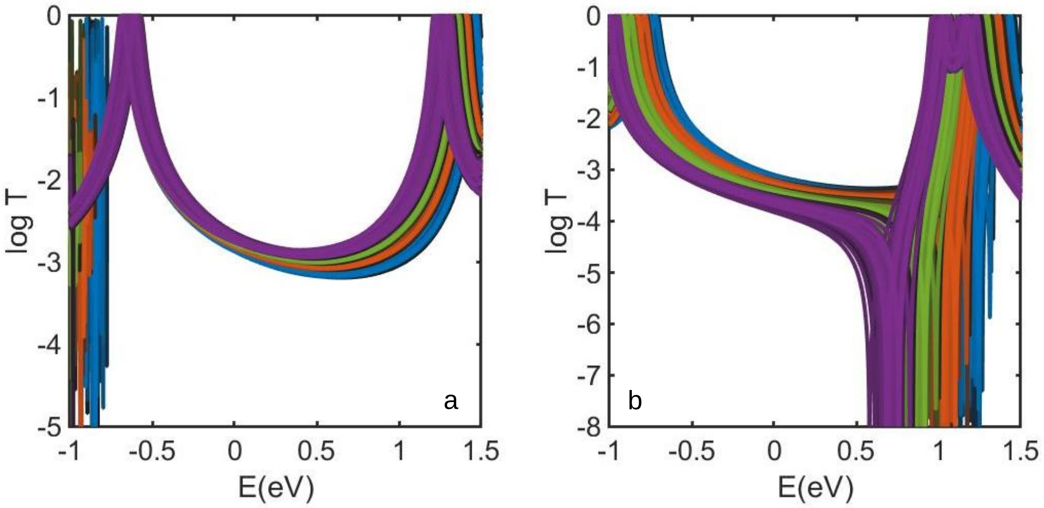

Figure S27: Individual TB transmission curves as a function of a random set of on-site energies in the range of $\pm 0.2\ eV$ on the system (a) when only the on-site energy of the chalcogen ε_C changes from -0.6, -0.8, -1, and -1.2 for O, S, Se, and Te, respectively, and (b) when the ε_C changes from -0.6, -0.8, -1, and -1.2 for O, S, Se, and Te, respectively and the ε_β are -0.48, -0.64, -0.83 and -1 for molecules **1**-**4**, respectively.

5.2 DFT Calculations

The optimized geometry and ground state Hamiltonian and overlap matrix elements of each structure (shown in Figure S22) was self-consistently obtained using the SIESTA[16,17] implementation of density functional theory (DFT). SIESTA employs norm-conserving pseudo-potentials to account for the core electrons and linear combinations of atomic orbitals to construct the valence states. The local density approximation (GGA) of the exchange and correlation functional is

used with CA parameterization, a double-ζ polarized (DZP) basis set, a real-space grid defined with an equivalent energy cut-off of 250 Ry. The geometry optimization for each structure is performed to the forces smaller than 10 meV/Å.

The mean-field Hamiltonian obtained from the converged DFT calculation was combined with the GOLLUM[13] implementation of the non-equilibrium Green's function method to calculate the phase-coherent, elastic scattering properties as discussed for the TB calculations.

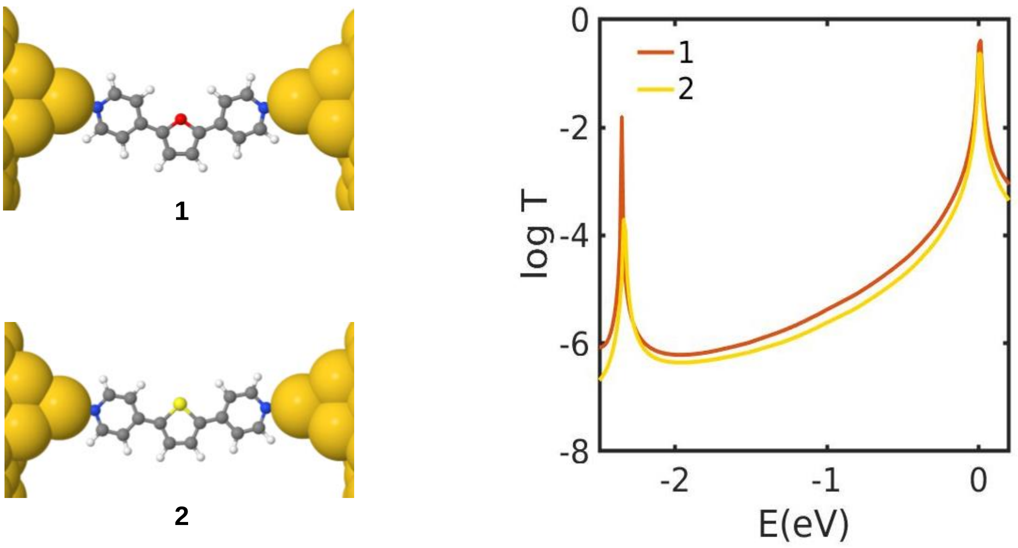

Figure S28: (a) Relaxed structure of the molecules **1** and **2** between Au electrodes. (b) DFT-based electrical transmission for corresponding structure.

References

(1) Faigl, F.; Deák, S.; Mucsi, Z.; Hergert, T.; Balázs, L.; Sándor, B.; Balázs, B.; Holczbauer, T.; Nyerges, M.; Mátravölgyi, B. A Novel and Convenient Method for the Preparation of 5-(Diphenylmethylene)-1H-Pyrrol-2(5H)-Ones; Synthesis and Mechanistic Study. *Tetrahedron* **2016**, *72* (35), 5444–5455. https://doi.org/10.1016/j.tet.2016.07.032.

(2) Merkul, E.; Urselmann, D.; Müller, T. J. J. Consecutive One-Pot Sonogashira–Glaser Coupling Sequence – Direct Preparation of Symmetrical Diynes by Sequential Pd/Cu Catalysis. *Eur. J. Org. Chem.* **2011**, *2011* (2), 238–242. https://doi.org/10.1002/ejoc.201001472.

(3) Sweat, D. P.; Stephens, C. E. A Modified Synthesis of Tellurophene Using NaBH4 to Generate Sodium Telluride. *J. Organomet. Chem.* **2008**, *693* (14), 2463–2464. https://doi.org/10.1016/j.jorganchem.2008.04.022.

(4) Bunzen, J.; Iwasa, J.; Bonakdarzadeh, P.; Numata, E.; Rissanen, K.; Sato, S.; Fujita, M. Self-Assembly of M24L48 Polyhedra Based on Empirical Prediction. *Angew. Chem. Int. Ed.* **2012**, *51* (13), 3161–3163. https://doi.org/10.1002/anie.201108731.

(5) Dolomanov, O. V.; Bourhis, L. J.; Gildea, R. J.; Howard, J. A. K.; Puschmann, H. OLEX2 : A Complete Structure Solution, Refinement and Analysis Program. *J. Appl. Crystallogr.* **2009**, *42* (2), 339–341. https://doi.org/10.1107/S0021889808042726.

(6) Sheldrick, G. M. SHELXT - Integrated Space-Group and Crystal-Structure Determination. *Acta Crystallogr. A* **2015**, *71* (1), 3–8. https://doi.org/10.1107/S2053273314026370.

(7) Sheldrick, G. M. Crystal Structure Refinement with SHELXL. *Acta Crystallogr. Sect. C Struct. Chem.* **2015**, *71* (Md), 3–8. https://doi.org/10.1107/S2053229614024218.

(8) Mészáros, G.; Li, C.; Pobelov, I.; Wandlowski, T. Current Measurements in a Wide Dynamic Range—Applications in Electrochemical Nanotechnology. *Nanotechnology* **2007**, *18* (42), 424004. https://doi.org/10.1088/0957-4484/18/42/424004.

(9) Nikoo, S.; Meister, P. J.; Hayward, J. J.; Gauld, J. W. An Assessment of Computational Methods for Calculating Accurate Structures and Energies of Bio-Relevant Polysulfur/Selenium-Containing Compounds. *Molecules* **2018**, *23* (12), 3323. https://doi.org/10.3390/molecules23123323.

(10) Fringuelli, F.; Marino, G.; Taticchi, A.; Grandolini, G. A Comparative Study of the Aromatic Character of Furan, Thiophen, Selenophen, and Tellurophen. *J. Chem. Soc. Perkin Trans. 2* **1974**, *2*, 332–337. https://doi.org/10.1039/p29740000332.

(11) Julg, A.; François, P. Recherches sur la géométrie de quelques hydrocarbures non-alternants: son influence sur les énergies de transition, une nouvelle définition de l'aromaticité. *Theor. Chim. Acta* **1967**, *8* (3), 249–259. https://doi.org/10.1007/BF00527311.

(12) Gordy, W. Dependence of Bond Order and of Bond Energy Upon Bond Length. *J. Chem. Phys.* **1947**, *15* (5), 305–310. https://doi.org/10.1063/1.1746501.

(13) Ferrer, J.; Lambert, C. J.; García-Suárez, V. M.; Manrique, D. Z.; Visontai, D.; Oroszlany, L.; Rodríguez-Ferradás, R.; Grace, I.; Bailey, S. W. D.; Gillemot, K.; Sadeghi, H.; Algharagholy, L. A. GOLLUM: A next-Generation Simulation Tool for Electron, Thermal and Spin Transport. *New J. Phys.* **2014**, *16*, 093029. https://doi.org/10.1088/1367-2630/16/9/093029.

(14) Sadeghi, H. Theory of Electron, Phonon and Spin Transport in Nanoscale Quantum Devices. *Nanotechnology* **2018**, *29* (37), 373001. https://doi.org/10.1088/1361-6528/aace21.

(15) Landauer, R. Spatial Variation of Currents and Fields Due to Localized Scatterers in Metallic Conduction. *IBM J. Res. Dev.* **1957**, *1* (3), 223–231. https://doi.org/10.1147/rd.13.0223.

(16) Artacho, E.; Anglada, E.; Diéguez, O.; Gale, J. D.; García, A.; Junquera, J.; Martin, R. M.; Ordejón, P.; Pruneda, J. M.; Sánchez-Portal, D.; Soler, J. M. The SIESTA Method; Developments and Applicability. *J. Phys. Condens. Matter Inst. Phys. J.* **2008**, *20* (6), 064208. https://doi.org/10.1088/0953-8984/20/6/064208.

(17) Soler, J. M.; Artacho, E.; Gale, J. D.; García, A.; Junquera, J.; Ordejón, P.; Sánchez-Portal, D. The SIESTA Method for Ab Initio Order- N Materials Simulation. *J. Phys. Condens. Matter* **2002**, *14* (11), 2745–2779. https://doi.org/10.1088/0953-8984/14/11/302.
